# Circulating exosomal microRNAs as potential prognostic biomarkers in gastrointestinal cancers: a systematic review and meta-analysis

**DOI:** 10.1186/s12935-023-02851-8

**Published:** 2023-01-20

**Authors:** Elmira Gheytanchi, Fatemeh Tajik, Mahdieh Razmi, Sadegh Babashah, William Chi Shing Cho, Kiarash Tanha, Maryam Sahlolbei, Roya Ghods, Zahra Madjd

**Affiliations:** 1 Oncopathology Research Center, Iran University of Medical Sciences, Tehran, Iran; 2 Department of Molecular Genetics, Faculty of Biological Sciences, Tarbiat Modares University, Tehran, Iran; 3 Department of Clinical Oncology, Queen Elizabeth Hospital, Kowloon, Hong Kong Special Administrative Region, China; 4 Department of Biostatistics, School of Public Health, Iran University of Medical Sciences, Tehran, Iran; 5 Department of Molecular Medicine, Faculty of Advanced Technologies in Medicine, Iran University of Medical Sciences, Tehran, Iran

**Keywords:** Circulating exosomal microRNAs (exomiRs), Gastrointestinal cancers, Prognostic value, Clinicopathological characteristics, Meta-analysis

## Abstract

**Background:**

Recent reports suggested that circulating exosomal microRNAs (exomiRs) may serve as non-invasive prediction biomarkers in gastrointestinal (GI) cancers, yet their clinicopathological and prognostic values need to be more clarified. Hence, the present meta-analysis was aimed to quantitatively assess the evidence regarding the association between circulating exomiRs and prognosis in GI cancer patients.

**Methods:**

A comprehensive search was carried out in prominent literature databases, including PubMed, ISI Web of Science, Scopus, and Embase. Odds ratios (ORs) or hazard ratios (HRs) with 95% confidence intervals (CIs) were gathered to evaluate the strength of the association. The quality assessment was investigated through the Newcastle-Ottawa Scale (NOS) and publication bias via Eggers’ test and funnel plots.

**Results:**

A total of 47 studies, comprising of 4881 patients, were considered eligible for this meta-analysis. Both up-regulated and down-regulated circulating exomiRs are significantly associated with differentiation (HR = 1.353, P = 0.015; HR = 1.504, P = 0.016), TNM stage (HR = 2.058, P < 0.001; HR = 2.745, P < 0.001), lymph node metastasis (HR = 1.527, P = 0.004; HR = 2.009, P = 0.002), distant metastasis (HR = 2.006, P < 0.001; HR = 2.799, P = 0.002), worse overall survival (OS) (HR = 2.053, P < 0.001; HR = 1.789, P = 0.001) and poorer disease/relapse/progression-free survival (DFS/RFS/PFS) (HR = 2.086, P < 0.001; HR = 1.607, P = 0.001) in GI cancer patients, respectively. In addition, subgroup analyses based on seven subcategories indicated the robustness of the association. The majority of findings were lack of publication bias except for the association between up-regulated exomiRs and OS or DFS/RFS/PFS and for the down-regulated exomiRs and TNM stage.

**Conclusion:**

This study supports that up- and down-regulated circulating exomiRs are associated with poorer survival outcomes and could be served as potential prognostic biomarkers in GI cancers. Given the limitations of the current findings, such as significant heterogeneity, more investigations are needed to fully clarify the exomiRs prognostic role.

**Supplementary Information:**

The online version contains supplementary material available at 10.1186/s12935-023-02851-8.

## Introduction

Gastrointestinal (GI) cancers, which include esophageal, gastric, pancreas, colorectal, rectal, and hepatocellular carcinomas, are the leading cause of cancer-related deaths, with incidence rates varying among industrialized and developing countries [1–4]. As reported in 2022, the number of new GI cancer cases and deaths were estimated at 343,040 and 171,920 in the USA, respectively [3]. Although the mortality rate of GI cancers has decreased over the last decade owing to multidisciplinary treatment approaches, the global burden of the disease remains considerable, with a notable unfavorable prognosis. The primary factors causing inferior survival outcomes for these individuals are late-stage diagnosis, inadequate prognostic biomarkers, metastasis and recurrence progression, and therapeutic resistance [5, 6].

The extent burden of GI cancers resulted in the planning of innovative molecular–omics landscapes [5–7]. Historically, tissue biopsy, as the standard method, still provides insight into cancer diagnosis and prognosis. However, this approach is invasive, costly, and associated with some complications, including a partial snapshot of the whole tumor, inaccessibility of tumor tissue in terms of anatomic location, biopsy sampling errors, and inter-observer variability in some GI tissues [8, 9]. Therefore, an accurate non-invasive detection technique is urgently warranted to completely elucidate the characteristics of the tumor, allow for early detection of cancer, and precisely evaluate the efficacy of treatment approaches. Compared to traditional tissue biopsy, blood-based or liquid biopsy as the minimally invasive tools provide close monitoring to identify cancer-associated changes and predict prognosis and acquired resistance or disease recurrence before the appearance of clinical symptoms [10–15].

Exosomes have received much attention in recent years as circulating biomarkers for cancer. Exosomes are cell-secreted nano-sized membrane (30 to 100 nm) vesicles involved in intercellular communication and various pathological features of cancers, including invasion, angiogenesis, immune response modulation, and inflammation [16, 17]. Extracellular vesicles (EVs), which may be recovered from a variety of physiological fluids, often reflect the genetic makeup of the cancer cells that originally made them up [19]. Importantly, microRNAs (miRs) in EVs or exomiRs obtained from blood samples demonstrate the specificity of tumors, indicating that exomiRs may be potential indicators for the diagnosis and prognosis of many malignancies as well as in the age of tailored anticancer therapy [17, 18, 20]. MiRs are endogenous non-coding RNAs subtype with 18–22 nucleotides which mainly modulate cellular gene expression, mostly at the post-transcriptional level. They are involved in many physiological cellular processes, including differentiation, proliferation, and apoptosis [18–21]. Recently, many investigations have reported that aberrant expression of miRs is closely associated with the progression of cancers [22, 23], suggesting miRs as putative biomarkers for diagnosis and prognosis in various tumors, such as colorectal cancer, non-small cell lung carcinoma (NSCLC), and glioma [24–26]. Non-exosomal and exosomal miRNAs (exomiRs) in body fluids are considered as stable "tumor-specific" circulating biomarkers in early diagnosis, prognosis, and screening of various cancer types, including colorectal, esophagus, and hepatocellular cancers [10, 11, 27, 28].

**Fig. 1:**
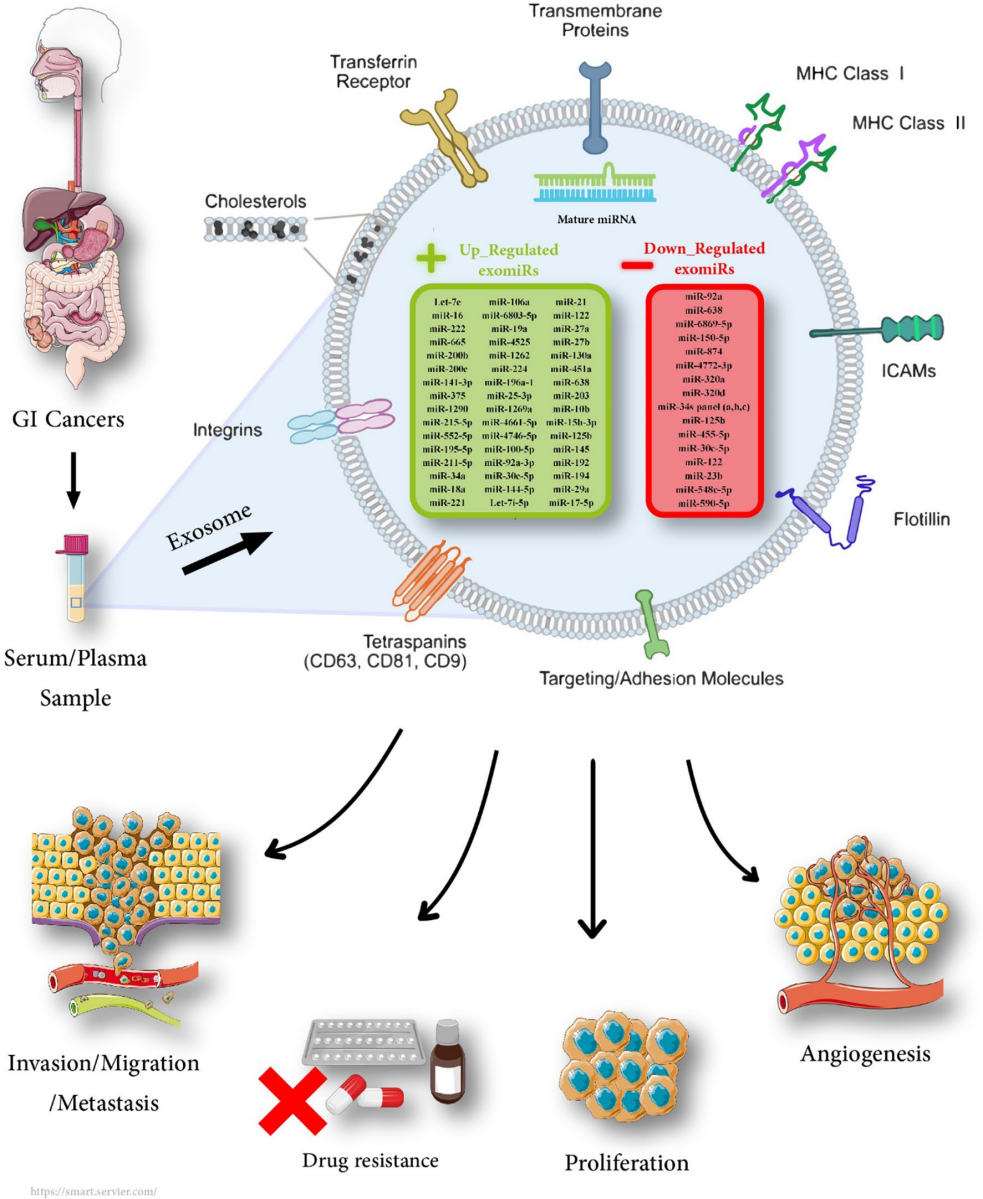
Deregulated circulating exosomal microRNA(s) in gastrointestinal cancers

This comprehensive systematic review and meta-analysis was designed to verify the prognostic significance of circulating exomiRs in patients with GI cancers and propose their potential as a non-invasive prognostic tool for monitoring mortality, focusing on clinicopathological outcomes. Deregulated circulating exosomal microRNA(s) in gastrointestinal cancers are shown in Fig. 1.

## Material and method

### Protocol and registration

This systematic review and meta-analysis has been registered in the PROSPERO International prospective register of systematic reviews (http://www.crd.york.ac.uk/PROSPERO), with the registration number: PROSPERO CRD42017057129; available at https://www.crd.york.ac.uk/prospero/display_record.php?RecordID=57129. In addition, the protocol of the current review has been published in the Systematic Reviews Journal [29].

### Eligibility criteria

The research question has been developed using PFO; “P” as Population, “F” as Prognostic Factors (or models of interest), and “O” as Outcome [30]. The following criteria were incorporated into this systematic review based on the PFO components:

- i) Observational studies (case-control, cross-sectional, and cohort studies) assessed the association between circulating exomiRs and GI cancers.
- ii) Studies published in English with available full texts.
- iii) Studies with human GI cancers, including upper and lower GI and hepatopancreatic biliary.
- iv) Studies assessing the association between the circulating exomiRs expression and the prognostic values consisting of overall survival (OS), disease/relapse/progression-free survival (DFS/RFS/PFS), and/or clinicopathological characteristics of GI cancers.
- v) Hazard ratios (HRs) and 95% confidence intervals (CIs) provided in the article, or availability of data to calculate HRs with 95% CIs.

The investigations meeting the following criteria were excluded:

- i) Reviews, meta-analysis, commentaries, case reports, case series studies.
- ii) In vitro and in vivo studies.
- iii) Studies not related to the topic of the interest (e.g., when the studies evaluated the other solid cancers).
- iv) Studies with insufficient and useless data or with unavailable full text.
- v) Studies in which cases received anti-cancer treatment (i.e., chemotherapy and/or radiation therapy) before the biopsy.

### Information sources

The literature was searched in electronic databases, comprising of Web of Science, PubMed/MEDLINE, Scopus, and Embase until 31st July 2017 and updated on 7th September 2022. Besides, the references of included papers were assessed. Hand searching was performed in key journals that rely on search in Scopus.

### Search strategy

This study was designed based on the Preferred Reporting Items for Systematic Reviews and Meta-Analyses guidelines (PRISMA 2020) [31]. The following main keywords were used to carry out the search strategy in the mentioned databases: (Neoplasms OR Cancer OR Carcinoma OR Tumor) AND (“Gastrointestinal Tracts” OR “GI Tract” OR “Digestive Tract”) AND (“extracellular vesicles” OR microvesicle OR “Shedding Microvesicles” OR exosomes). The search syntax was adopted in other databases. The search strategy has been fully presented in Additional file 3: Table S1.

### Selection process

Studies were selected in three phases. Phase 1: duplicated studies were deleted using both EndNote software (version X9.3.3, Thomson Reuters, Philadelphia, USA) and hand searching. Phase 2: two authors (E.Gh. and F.T.) independently screened all records by title and abstract. Phase 3: the same authors independently assessed the full text of each potentially eligible study. Any disagreement was resolved through consensus and then checked by a third author (M.R.).

### Data collection process

The data of each eligible study’ was extracted by two authors (E.Gh. and F.T.) independently. The obtained data were entered into a “Data Extraction Form” created by Microsoft Excel for quality assessment and data synthesis. A consensus method was applied between the two reviewers to finalize the validity of all collected data and was then checked by a third author (M.R.).

### Data items

Data extracted from all eligible papers have consisted of the following items: author’s name, publication year, country, type of exomiRs, type of cancer, expression of exomiRs, detection method, exosome extraction method, sample size, sample type, age, gender, number of patients with up- and down-regulation of circulating exomiRs, clinicopathological parameters (gender, TNM stage of disease, tumor differentiation, lymph node metastasis, distant metastasis), survival data (HR with corresponding 95% CI for OS and DFS/RFS/PFS), cut-off value, and median or mean follow-up times.

### Study quality assessment

All studies reporting the prognostic values of exomiRs were included in the meta-analysis and assessed according to Newcastle–Ottawa Scale (NOS) tool by two independent investigators (E.Gh. and F.T.). NOS comprises three sections: selection, comparability, and exposure or outcome, with a score ranging from 0 to 9 [32]. This scoring includes four stars for the selection section, two stars for comparability, and three stars for exposure or outcomes. The result of the quality assessment is divided into three categories good, fair, and poor. Moreover, discrepancies between the two authors were resolved by consensus and were then checked by a third author (M.R.).

### Effect measures and synthesis methods

Comprehensive Meta-Analysis (CMA) software version 2.2.064 was applied to perform all statistical analyses. The HRs and corresponding 95% CIs were recorded for all survival data, including OS and DFS/RFS/PFS. HRs were extracted from both multivariate and univariate statistical tests by preferring information from multivariate statistics if available. For studies without providing HR, we calculated HRs by the Kaplan–Meier curves using the method presented by Parmar et al. [33]. In this regard, survival data were extracted from Kaplan-Meyer curves by the software GetData Graph Digitizer (http://getdata-graph-digitizer.com/). Additionally, the combined odds ratios (ORs) and 95% CIs were applied to evaluate the associations between exomiRs expression and clinicopathological features, including gender (male vs. female), TNM stage (III/IV vs. I/II), tumor differentiation (poor vs. moderate/well), lymph node metastasis (positive vs. negative) and distant metastasis (positive vs. negative). A pooled HR/OR larger than one reflected a worse clinical prognostic outcome in GI cancer patients. Heterogeneity among studies was assessed through Cochran’s Q statistic and the I^2^ index. While an I^2^ of over 50% and/or P < 0.05 indicated a large degree of heterogeneity and a random effect model was used, the fixed effect model was utilized in the absence of heterogeneity (I^2^ ≤ 50% or P > 0.05). Afterward, subgroup analyses were employed for prognostic outcomes to recognize possible causes of heterogeneity and evaluate the prognostic importance of various subgroups. The level of significance was set at P < 0.05.

### Publication bias assessment

Funnel plots were applied to graphically investigate the potential publication bias. In addition, Egger’s test was conducted to statistically assess the publication bias [34].

## Results

### Study selection

**Fig. 2:**
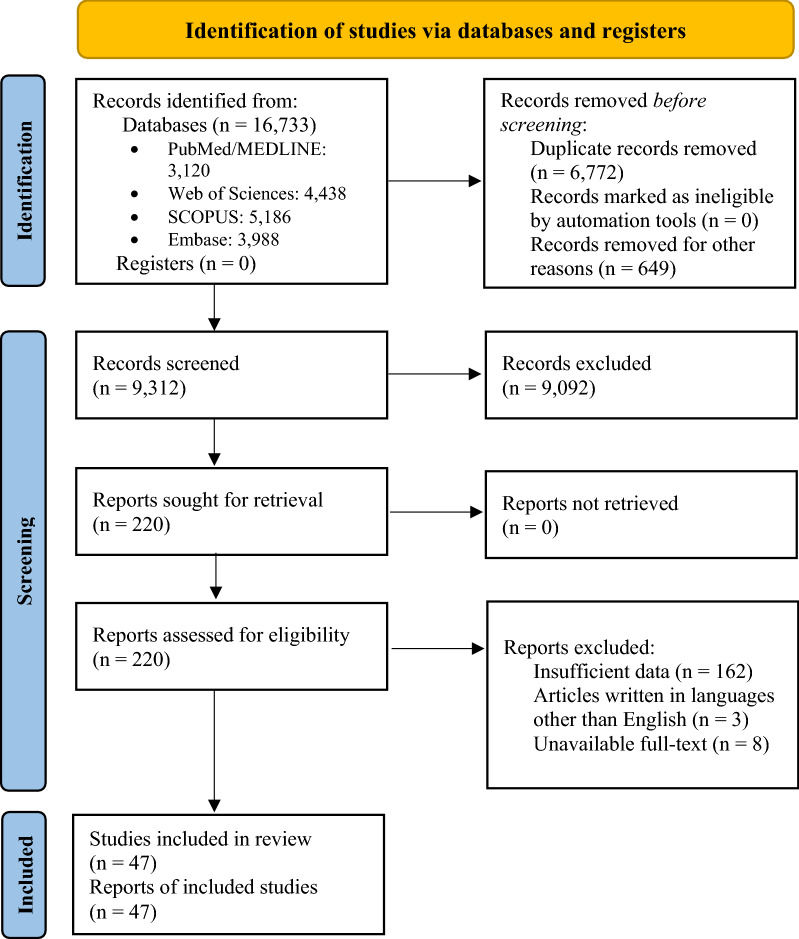
Flow-chart for the search strategy according to the Preferred Reporting Items for Systematics Reviews and Meta-Analyses (PRISMA) guideline

The preliminary search contained 16,733 references, of which 3120, 4438, 5186, and 3988 papers were retrieved from PubMed/MEDLINE, Web of Science, Scopus, and Embase databases, respectively, published from inception to 7th September 2022. Subsequently, the resulting references were imported to the EndNote reference manager to remove duplicate articles (n = 6772). The review articles were excluded (n = 649) before screening studies. Of the remained articles (n = 9312), 9092 papers were excluded following the subsequent screening of titles and abstracts according to eligibility criteria. As a result, 220 eligible studies remained and entered the next phase. The full text of the remaining studies was evaluated, and of these, 173 were excluded according to the exclusion criteria and unavailable full text. Finally, 47 studies were included in the qualitative synthesis. A flowchart of the search process for eligible studies is shown in Fig. 2.

### Study characteristics

All the enrolled studies were written in English and published between 2015 and 2022, with sample sizes ranging from 4 to 326 patients (Table 1). Geographically, the majority of the papers (n = 31) were conducted in China, whereas the remaining papers (n = 16) were carried out in other countries (Japan, Egypt, Korea, Spain, Germany, Norway, and Taiwan). In addition, a large number of cases were male in most of the studies. Of 47 studies, 32 reported circulating exomiRs with high aberrant expression, and 16 reported low expression (Additional file 4: Table S2). There have been various circulating exomiRs, leading to high heterogeneity in our study. Concerning the types of cancer, the two most commonly evaluated GI tract carcinomas were colorectal cancer (CRC) (n = 16) and hepatocellular carcinoma (HCC) (n = 16), followed by gastric cancer (GC) (n = 8), pancreatic ductal adenocarcinoma (PDAC) (n = 3), hepatoblastoma (HB) (n = 2), pancreatic cancer (PC) (n = 1), and locally-advanced rectal cancer (LARC) (n = 1). Moreover, the exomiRs were derived from either serum (n = 32) or plasma (n = 15). Most of the articles (n = 38) used quantitative real-time PCR (qRT-PCR) for the detection of exomiR expression; 7 studies employed reverse transcription quantitative PCR (RT-qPCR), and 3 applied RNA sequencing. There have been multiple exosome isolation methods; 28 studies employed extraction kit, and 19 papers applied ultracentrifuge (UC). Besides, 33 studies evaluated the relationship between OS and the expression of circulating exomiRs, while 25 studies investigated the prognostic value of the circulating exomiRs on DFS/RFS.

**Table 1:** Main characteristics of the studies included in the meta-analysis

Authors, publication year	Country, Duration (year)	Exosomal miR(s)	Cancer type	Detection method	Exosome extraction method	sample	Sample size (cancer)	mean/ median age (year)	Gender cancer (M/F)	TNM Stage	Outcome	Hazard ratio (HR)	Follow up (month)	Spec %	Sen %	AUC (95% CI)	NOS
Up-regulation^a^																	
Soeda N 2019 [35]	Japan 2006–2015	miR-21	GC	qRT-PCR	UC	plasma	129	68	90/39	II—III	OS, RFS	OS: R-Multi/ R-Uni RFS: R-Multi/ R-Uni	40.8	76.8	61.6	NA	good
Sun L 2020 [36]	China NA	miR-122	CRC	qRT-PCR	UC	serum	85	65	46/39	I—IV	OS	R-Multi/ R-Uni	60	NA	NA	NA	poor
Liu X 2018 [37]	China NA	miR-27a, miR-130a	CRC	qRT-PCR	Exosome isolation kit	plasma	130	64	80/50	I—IV	OS	R-Uni	60	90.9 100	81.8 69.3	0.866 (0.774–0.957) 0.816 (0.730–0.901)	poor
Wu L. 2020 [38]	China 2018–2019	miR-21 miR-210	PC	RT-qPCR	Exosome isolation kit	serum	30	60	20/10 23/7	I—IV	NA	NA	NA	90.0 90.0	80.0 83.0	0.869 0.823	good
Takahasi K 2018 [39]	Japan 2013- 2017	miR-451a	PDAC	qRT-PCR	UC	plasma	50	High expression group: 66 Low expression group: 69	28/22	I—II	OS, DFS	OS: R-Multi/ R-Uni DFS: R-Multi/ R-Uni	30 (range: 5.5–54)	70.8	69.2	NA	good
Tsukamoto M 2017 [40]	Japan 2002- 2012	miR-21	CRC	RT-qPCR	UC	plasma	326	NA	NA	I—IV	OS, DFS	OS: R-Multi DFS: R-Multi	55	NA	NA	NA	poor
Yokota Y 2021 [41]	Japan 2012–2015	miR-638	HCC	RT-qPCR	Exosome isolation kit	serum	54	High expression group: 73 Low expression group: 70	35/19	NA	DFS	R-Multi/ R-Uni	24	NA	NA	NA	good
Takano Y 2017 [42]	Japan 2005- 2012	miR-203	CRC	RT-qPCR	UC	serum	240	NA	147/93	I—IV	OS, DFS	OS: R-Multi/ R-Uni DFS: R-Multi/ R-Uni	54	NA	NA	NA	poor
Tian X 2019 [43]	China 2009–2012	miR-21 miR-10b	HCC	qRT-PCR	ExoQuick kit	serum	124	48	115/9	NA	DFS	R-Multi/ R-Uni	NA	NA	NA	NA	poor
Wei S.C. 2020 [44]	China NA	miR-15b-3p	GC	qRT-PCR	UC	serum	108	62	80/28	I—IV	OS	E	NA	80.6	74.1	0.820 (0.763–0.876)	good
Xue X. F. 2019 [45]	China 2015–2017	miR-122 miR‐125b miR‐145 miR‐192 miR‐194 miR‐29a miR‐17‐5p miR‐106a	HCC	qRT-PCR	Exosome isolation kit	serum	80	59	61/19	I—IV	OS	E	24	NA	NA	0.746 (0.650–0.842) 0.650 (0.526–0.774) 0.535 (0.422–0.649) 0.752 (0.658–0.846) 0.738 (0.638–0.838) 0.703 (0.597–0.809) 0.850 (0.764–0.936) 0.704 (0.534–0.873)	good
Yagi T 2019 [46]	Japan NA	miR-125b	CRC	qRT-PCR	UC	plasma	6	65	4,2	III—IV	PFS	R-Multi/ R-Uni	12	NA	NA	NA	good
Yan S. S. 2018 [47]	China NA	miR-6803-5p	CRC	qRT-PCR	Exosome isolation kit	serum	168	55	100/68	I—IV	OS, DFS	OS: R-Multi DFS: R-Multi	60	NA	NA	0.739	poor
Matsumura T. 2015 [48]	Japan 1992–2007	miR-19a	CRC	qRT-PCR	UC	serum	209	65	80/129	I—IV	OS, DFS	OS: R-Multi/ R-Uni DFS: R-Multi/ R-Uni	55.68	NA	NA	NA	poor
Kawamura S. 2019 [49]	Japan 2013- 2018	miR-4525 miR-451a miR-21	PDAC	qRT-PCR	UC	plasma	55	miR-4525: 66 miR-451a: 69 miR-21: 70	33/22	I—II	OS, DFS	OS: R-Multi/ R-Uni DFS: R-Multi/ R-Uni	60	86.4 77.3 72.7	81.8 72.7 72.7	NA	poor
Abd El Gwad A. 2018 [50]	Egypt 2015–2016	miR-1262	HCC	qRT-PCR	UC	serum	60	58	43/17	N/A	RFS	E	44	80.0	95.0	0.847	good
Cui Y. 2019 [51]	China 2014–2016	miR-224	HCC	qRT-PCR	Exosome isolation kit	serum	89	59	43/46	N/A	OS	E	NA	NA	NA	0.910 (0.840–0.980)	fair
Feng C. 2019 [52]	China NA	miR-196a-1	GC	qRT-PCR	Exosome isolation kit	plasma	86	NA	NA	I—IV	OS	E	NA	NA	NA	NA	poor
Liu W. B. 2016 [53]	China 2008–2013	miR-21	HB	qRT-PCR	ExoQuick kit	serum	32	NA	17/15	I—IV	DFS	R-Multi/ R-Uni	42	NA	NA	0.861 (0.752–0.935)	poor
Cho H. J. 2020 [54]	Korea 2014–2018	miR-25-3p miR-1269a miR-4661-5p miR-4746-5p	HCC	Cohoort1: NGS-RNA-Seq Cohort 2&3: qRT-PCR	ExoQuick kit	serum	72	55	58/14	I—IV	NA	NA	NA	71.4	38.0	0.704 (0.629–0.772)	good
Han J. Y. 2020 [55]	China 2015–2017	hsa-miR-100-5p hsa-miR-92a-3p hsa-miR-30e-5p hsa-miR-144-5p hsa-let-7i-5p has-miR-16	CRC	qRT-PCR	Exosome isolation kit	plasma	139	60	77/62	III—IV	OS	R-Uni	NA	NA	NA	0.637 (0.545 -0.729) 0.659 (0.568–0.751) 0.694 (0.606–0.782) 0.791 (0.718 -0.864) 0.650 (0.559–0.742) 0.721 (0.638–0.804) 0.746 (0.664–0.827) 0.778 (0.702–0.854)	good
de Miguel-Pérez D 2020 [56]	Spain NA	miR-92a miR-222	CRC	qRT-PCR	UC	serum	44	55	30/14	NA	OS, DFS/PFS	miR-92a OS: R-Uni DFS/PFS: R-Multi/ R-Uni miR-222 OS: R-Multi/ R-Uni	27	NA	NA	0.951 (0.900–1.000) 0.896 (0.810–0.980)	good
Qu Z. 2017 [57]	China NA	miR-665	HCC	qRT-PCR	ExoQuick kit	serum	30	60	12/18	I—IV	OS	E	NA	NA	NA	NA	poor
Reese M. 2020 [58]	Germany 2015–2018	miR-200b miR-200c	PDAC	qRT-PCR	UC	serum	56	60	36/20	I—IV	OS	miR-200b OS: R-Multi/ R-Uni miR-200c OS: R-Multi/ R-Uni	13	NA	NA	0.790 (0.680–0.890) 0.670 (0.550–0.790)	good
Meltzer S. 2019 [59]	Norway NA	miR-141-3p miR-375	LARC	qRT-PCR	Exosome isolation kit	plasma	64	60	36/28	NA	OS, PFS	OS: R-Uni PFS: R-Uni	65 (range 4–66)	NA	NA	NA	good
Wang Q. 2021 [60]	China NA	miR-1290	HCC	qRT-PCR	Exosome isolation kit/UC	serum	49	55	37/12	I—IV	NA	NA	NA	NA	NA	NA	poor
Zhang Y. 2021 [60]	China NA	miR-215-5p	GC	qRT-PCR	ExoQuick kit	serum	118	60	70/48	I—IV	OS, DFS	OS: R-Multi/ R-Uni DFS: E	NA	97.1	68.6	0.866	good
Zhu L. 2022 [61]	China 2020–2021	miR-552-5p	GC	qRT-PCR	UC	plasma	30	56	16/14	I—IV	NA	NA	NA	NA	NA	NA	good
Yang J. 2022 [62]	China NA	miR-195-5p miR-211-5p	GC	RT-qPCR	UC	plasma	108	62.4 ± 8.9 62.2 ± 9.4	80/28 79/27	I—IV	NA	NA	NA	NA	NA	0.745 (0.584–0.906) 0.798 (0.656–0.940)	good
Chen S. 2022 [63]	China NA	miR-34a	HCC	qRT-PCR	UC	serum	60	55	60/60	I—IV	OS	R-Multi/ R-Uni	6–60	51.7	78.3	0.664 (0.572–0.747)	good
Huang C. Y. 2021[64]	China 2017–2018	let-7e miR-18a miR-27b miR-221 miR-20b miR-652	HCC	RT-qPCR	UC	plasma	40	68	13/7	NA	OS, DFS	OS: R-Uni DFS: R-Uni	80	NA	NA	NA	poor
Hao Y. J. 2022 [65]	Taiwan 2019–2020	miR-21	CRC	qRT-PCR	Exosome isolation kit	plasma	113	65	77/36	I—IV	DFS, PFS	E	NA	NA	NA	NA	poor
Down-regulation^a^																	
Soeda N 2019 [35]	Japan 2006–2015	miR-92a	GC	qRT-PCR	UC	plasma	129	68	90/39	II—III	OS, RFS	OS: R-Multi/ R-Uni RFS: E	40.8	60.7	63.0	NA	good
Yan S. 2017 [66]	China 2008–2014	miR-638	CRC	qRT-PCR	Exosome isolation kit	serum	192	58	108/84	I—IV	OS, DFS	OS: R-Multi/ R-Uni DFS: E	47	NA	NA	NA	poor
Yan S. S. 2018 [67]	China 2012–2014	miR-6869-5p	CRC	qRT-PCR	Exosome Isolation Kit	serum	142	59	85/57	I—IV	OS	R-Multi/ R-Uni	36	NA	NA	NA	good
Zou S. L. 2019 [68]	China NA	miR-150-5p	CRC	qRT-PCR	ExoQuick kit	serum	133	60	49/84	I—IV	OS, DFS	OS: R-Multi/ R-Uni DFS: E	60	76.1	81.0	0.870	poor
Zhang N. 2020 [69]	China NA	miR-874	CRC	RT-qPCR	Exosome Isolation kit	serum	125	60	76/49	I—IV	OS	R-Multi/ R-Uni	NA	78.6	80.8	0.818	poor
Liu C. 2016 [70]	China 2006–2011	miR-4772-3p	CRC	qRT-PCR RNA-seq	ExoQuick kit	serum	84	57	53/31	II—III	OS	R-Multi	51 (range: 45–64)	77.1	78.6	0.720 (0.590–0.850)	poor
Hao X. 2020 [71]	China 2012–2013	miR-320a	HCC	qRT-PCR	ExoQuick kit	serum	104	60	77/27	I—IV	OS, DFS	OS: R-Multi/ R-Uni DFS: E	NA	80.0	77.9	0.860	good
Jiao C. W. 2017 [72]	China 2007–2015	miR-34 s panel miR-34a miR-34b miR-34c	HB	qRT-PCR	ExoQuick kit	serum	89	NA	52/37	I—IV	DFS	R-Multi/ R-Uni	54	NA	NA	0.831(0.071–0.371) 0.813(0.023–0.296) 0.837(0.004–0.342)	good
Li W. 2020 [73]	China NA	miR-320d	HCC	qRT-PCR	Exosome isolation kit	serum	110	60	98/12	I—IV	OS, DFS	OS: R-Multi DFS: E	NA	NA	NA	0.869	good
Liu W. F. 2017 [74]	China 2012	miR-125b	HCC	qRT-PCR	ExoQuick kit	serum	128	50	110/18	I—III	OS	R-Multi/ R-Uni	2.9–52.4	53.4	82.5	0.702 (0.602–0.802)	good
Sheng L. Q. 2020 [75]	China 2017–2018	miR-455-5p miR-30c-5p	HCC	exomiRs sequencing	Exosome isolation kit	plasma	cohort1:24 cohort2:27	NA	cohort1:36/8 cohort2:8/4	N/A	OS	E	NA	NA	NA	NA	poor
Shi M. 2018 [76]	China 2008–2011	miR-638	HCC	qRT-PCR	Exosome isolation kit	serum	126	65	70/56	I—IV	OS	R-Multi/ R-Uni	81.5	NA	NA	NA	good
Suehiro T. 2018 [76]	Japan 2006–2013	miR-122	HCC	qRT-PCR	ExoQuick kit	serum	75	73	49/26	NA	DFS	R-Multi	47	NA	NA	NA	poor
Kumata Y. 2018 [77]	Japan 2006–2013	miR-23b	GC	qRT-PCR	UC	plasma	232	NA	165/67	I—IV	OS, DFS	OS: R-Multi/ R-Uni DFS: R-Multi/ R-Uni	45.6 (range: 4.8–127.2)	NA	NA	NA	poor
Peng Z. Y. 2018 [78]	China 2008–2014	miR-548c-5p	CRC	qRT-PCR	Exosome isolation kit	serum	108	58	61/47	I—IV	OS	R-Multi/ R-Uni	44	NA	NA	NA	poor
Zheng G-D. 2021 [79]	China 2008–2011	miR-590-5p	GC	qRT-PCR	UC	serum	168	60	117/51	I—IV	OS	R-Multi/ R-Uni	64.2 (range: 43.3–92)	86.0	63.7	0.810 (0.751–0.860)	good

*GC* Gastric Cancer, *CRC* Colorectal Cancer, *LARC* Locally Advanced Rectal Cancer, *HCC* Hepatocellular Carcinoma, *HB* Hepatoblastoma, *PC* Pancreatic Cancer, *PDAC* Pancreatic Ductal Adenocarcinoma, *UC* Ultracentrifuge, *microRNA* miR, *NA* Not Applicable, *OS* Overall Survival, *DFS* Disease Free Survival, *PFS* Progression Free Survival, *RFS* Relapse Free Survival, *E* Estimated, *R-Multi* Reported Multivariate, *R-Uni* Reported Univariate, *qRT-PCR* quantitative real-time PCR, *RT-qPCR* Reverse transcription quantitative PCR, *Sen* Sensitivity, *Spec* Specificity, *AUC* Area Under Curve.

^a^The up- and down-regulation of exomiR(s) has been reported in comparison with the control group

### Quality assessment in studies

**Fig. 3:**
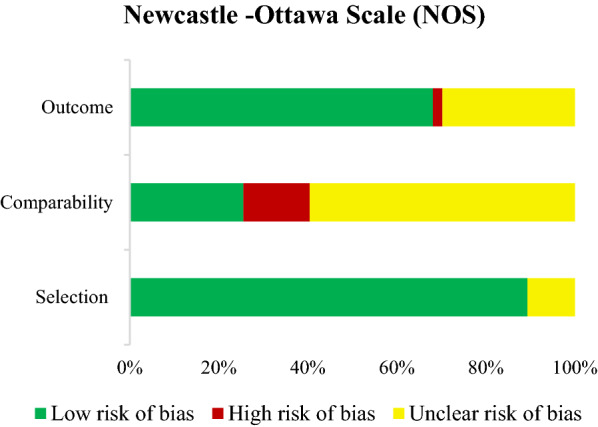
Summary of quality assessment

According to the NOS quality assessment tool, the majority of the included studies (n = 28) had good quality. The remaining studies (n = 18) had poor quality, and only one study had fair quality. A summary of quality assessment for all eligible studies has been shown in Fig. 3.

### Meta-analysis

#### Prognostic accuracy and subgroup analyses

A total of 34 studies included in the present meta-analysis assessed the association between deregulated exomiRs (n = 52) and OS in individuals suffering from GI cancers. Twenty-four studies provided data regarding exomiRs deregulation (n = 29) and DFS/RFS/PFS.

#### Deregulated exomiRs and the overall survival (OS)

**Fig. 4:**
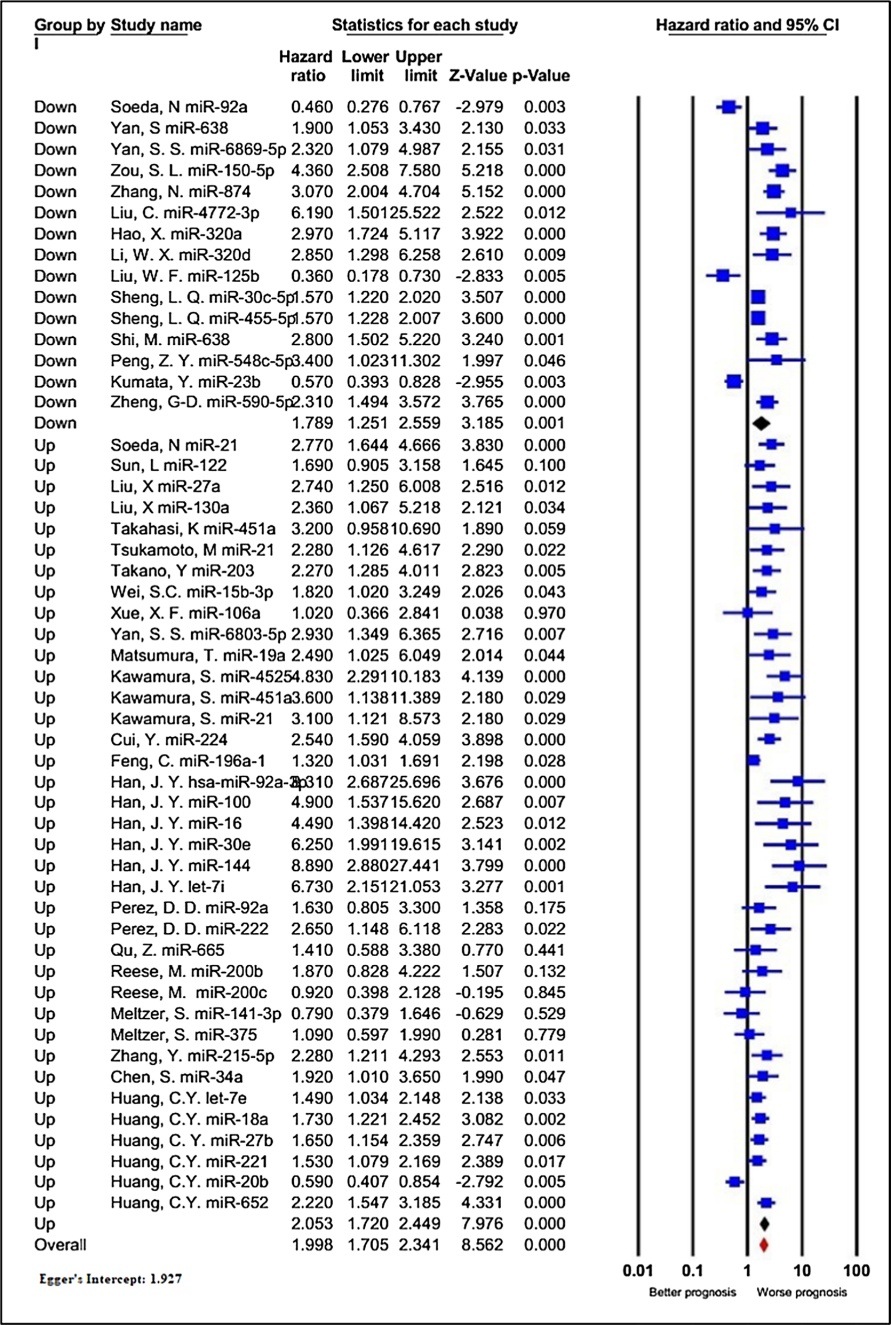
Forest plot of the association between exomiRs deregulation and overall survival in patients with GI cancers, stratified by the type of exomiRs deregulation (down-regulated exomiRs and up-regulated exomiRs)

Thirty-four studies containing 3833 patients investigated the impact of exomiRs deregulation on OS in patients with GI cancers. As shown in Table 2, since statistical heterogeneity was identified among the investigations (I^2^ = 76.564%, P < 0.001), a random-effect model was applied to estimate the combined HR. Patients with deregulated exomiRs displayed a statistically significant decrease in OS (pooled HR = 1.998, 95% CI 1.705–2.341, P < 0.001, Fig. 4).

**Table 2:** The results of meta-analyses for the association between deregulated exomiRs and overall survival (OS), and disease/relapse/progression-free survival (DFS/RFS/PFS) in patients with GI cancers

Study groups	Included exomiRs	Test of association		Test of heterogeneity	
		HR (95% CI)	P-value	I^2^%	P-value
OS					
All studies	52	1.998 (1.705–2.341)	< 0.001	76.564	< 0.001
The type of exomiRs deregulation					
Up-regulation	37	2.053 (1.720–2.449)	< 0.001	66.043	< 0.001
Down-regulation	15	1.789 (1.251–2.559)	0.001	87.290	< 0.001
Type of cancer					
Colorectal	21	2.928 (2.417–3.547)	< 0.001	19.747	0.204
Gastric	7	1.353 (0.827–2.214)	0.229	88.803	< 0.001
Hepatocellular	16	1.582 (1.264–1.979)	< 0.001	76.367	< 0.001
Pancreatic	6	2.514 (1.478–4.279)	0.001	48.243	0.085
locally advanced rectal	2	0.958 (0.601–1.525)	0.856	0.000	0.506
Sample size					
≥ 100	29	2.306 (1.789–2.973)	< 0.001	81.157	< 0.001
< 100	23	1.659 (1.359–2.025)	< 0.001	65.182	< 0.001
Sample type					
Plasma	27	1.890 (1.497–2.386)	< 0.001	82.109	< 0.001
Serum	25	2.125 (1.752–2.578)	< 0.001	51.047	0.002
Survival analysis					
Direct	44	2.114 (1.719–2.601)	< 0.001	79.360	< 0.001
Indirect	8	1.559 (1.368–1.776)	< 0.001	1.170	0.420
NOS score					
≥ 7	25	2.165 (1.577–2.972)	< 0.001	78.093	< 0.001
< 7	27	1.850 (1.534–2.231)	< 0.001	74.863	< 0.001
Ethnicity					
Asian	46	2.085 (1.746–2.489)	< 0.001	78.377	< 0.001
Caucasian	6	1.327 (0.934–1.886)	0.115	25.255	0.245
DFS/RFS/PFS					
All studies	29	1.920 (1.641–2.245)	< 0.001	55.871	< 0.001
Deregulation					
Up-regulation	21	2.086 (1.725–2.522)	< 0.001	41.988	0.023
Down-regulation	8	1.607 (1.218–2.122)	0.001	72.320	0.001
Type of cancer					
Colorectal	9	2.105 (1.736–2.554)	< 0.001	0.000	0.734
Gastric	4	1.408 (0.781–2.539)	0.256	86.773	< 0.001
Hepatocellular	10	1.923 (1.651–2.239)	< 0.001	0.000	0.740
Pancreatic	4	3.195 (2.019–5.056)	0.000	0.000	0.839
Locally advanced rectal	2	1.034 (0.758–1.410)	0.834	0.000	0.567
Sample size					
≥ 100	15	1.911 (1.520–2.402)	< 0.001	64.800	< 0.001
< 100	14	1.890 (1.506–2.373)	< 0.001	45.085	0.034
Sample type					
Plasma	13	1.819 (1.317–2.510)	< 0.001	74.592	< 0.001
Serum	16	1.906 (1.680–2.163)	< 0.001	0.000	0.633
Survival analysis					
Direct	20	2.019 (1.594–2.558)	< 0.001	67.219	< 0.001
Indirect	9	1.809 (1.552–2.109)	< 0.001	0.000	0.725
NOS score					
≥ 7	14	1.803 (1.498–2.170)	< 0.001	41.843	0.050
< 7	15	2.062 (1.571–2.706)	< 0.001	65.905	< 0.001
Ethnicity					
Asian	26	1.998 (1.705–2.341)	< 0.001	50.425	0.002
Caucasian	3	1.109 (0.800–1.538)	0.535	10.141	0.329

Additionally, to figure out the overall findings’ robustness, subgroup analysis for OS data was conducted according to seven subcategories, including the type of exomiRs deregulation (up or down), type of cancer, sample size, different data extraction methods (direct or indirect), NOS score, and ethnicity (Table 2). Stratified analysis by the different types of exomiRs deregulation indicated a poorer OS for 37 up-regulated exomiRs (HR = 2.053, 95% CI 1.720–2.449, P < 0.001; I^2^ = 66.043%, P < 0.001), compared to 15 down-regulated exomiRs (HR = 1.789, 95% CI 1.251–2.559, P = 0.001; I^2^ = 87.290%, P < 0.001). Regarding the cancer types, deregulation of exomiRs was closely associated with inferior OS in cases with CRC (HR = 2.928, 95% CI 2.417–3.547, P < 0.001, I^2^ = 19.747%, P = 0.204), HCC (HR = 1.582, 95% CI 1.264–1.979, P < 0.001, I^2^ = 76.367%, P < 0.001), and PDAC (HR = 2.514, 95% CI 1.478–4.279, P = 0.001, I^2^ = 48.243%, P = 0.085). However, GC (HR = 1.353, 95% CI 0.827–2.214, P = 0.229, I^2^ = 88.803%, P < 0.001) and LARC (HR = 0.958, 95% CI 0.601–1.525, P = 0.856, I^2^ = 0.000%, P = 0.506) did not show such association. When the investigations were stratified based on the sample size, a more considerable relationship was identified between worse OS and large sample sizes (≥ 100, HR = 2.306, 95% CI 1.789–2.973, P < 0.001; I^2^ = 81.157%, P < 0.001) compared to small sample sizes (< 100, HR = 1.659, 95% CI 1.359–2.025, P < 0.001; I^2^ = 65.182%, P < 0.001). Subgroup analysis of OS for exomiRs de-regulation in plasma demonstrated considerable association (HR = 1.890, 95% CI 1.497–2.386, P < 0.001; I^2^ = 82.109%, P < 0.001), like serum (HR = 2.125, 95% CI 1.752–2.578, P < 0.001; I^2^ = 51.047%, P = 0.002). In subgroup analysis stratified by different data extraction methods, exomiRs deregulation revealed a more significant connection with the worse OS in the HR presented in the articles (HR = 2.114, 95% CI 1.719–2.601, P < 0.001; I^2^ = 79.360%, P < 0.001) than that calculated from the survival curves (HR = 1.559, 95% CI 1.368–1.776, P < 0.001; I^2^ = 1.170%, P = 0.420). When categorized by quality assessment, deregulated exomiRs was related to poorer OS in both high- (HR = 2.165, 95% CI 1.577–2.972, P < 0.001; I^2^ = 78.093%, P < 0.001) and low-quality publications (HR = 1.850, 95% CI 1.534–2.231, P < 0.001; I^2^ = 74.863%, P < 0.001) (Additional file 1: Figure S1). Finally, in subgroup analysis stratified by ethnicity, deregulation of exomiRs revealed a significant connection with the worse OS in the articles published in Asian countries (HR = 2.085, 95% CI 1.746–2.489, P < 0.001; I^2^ = 78.377%, P < 0.001) unlike articles from Caucasian countries with an HR of 1.327 (95% CI 0.934–1.886, P = 0.115; I^2^ = 25.255%, P = 0.245).

#### Deregulated exomiRs and the disease/relapse/progression-free survival (DFS/RFS/PFS)

**Fig. 5:**
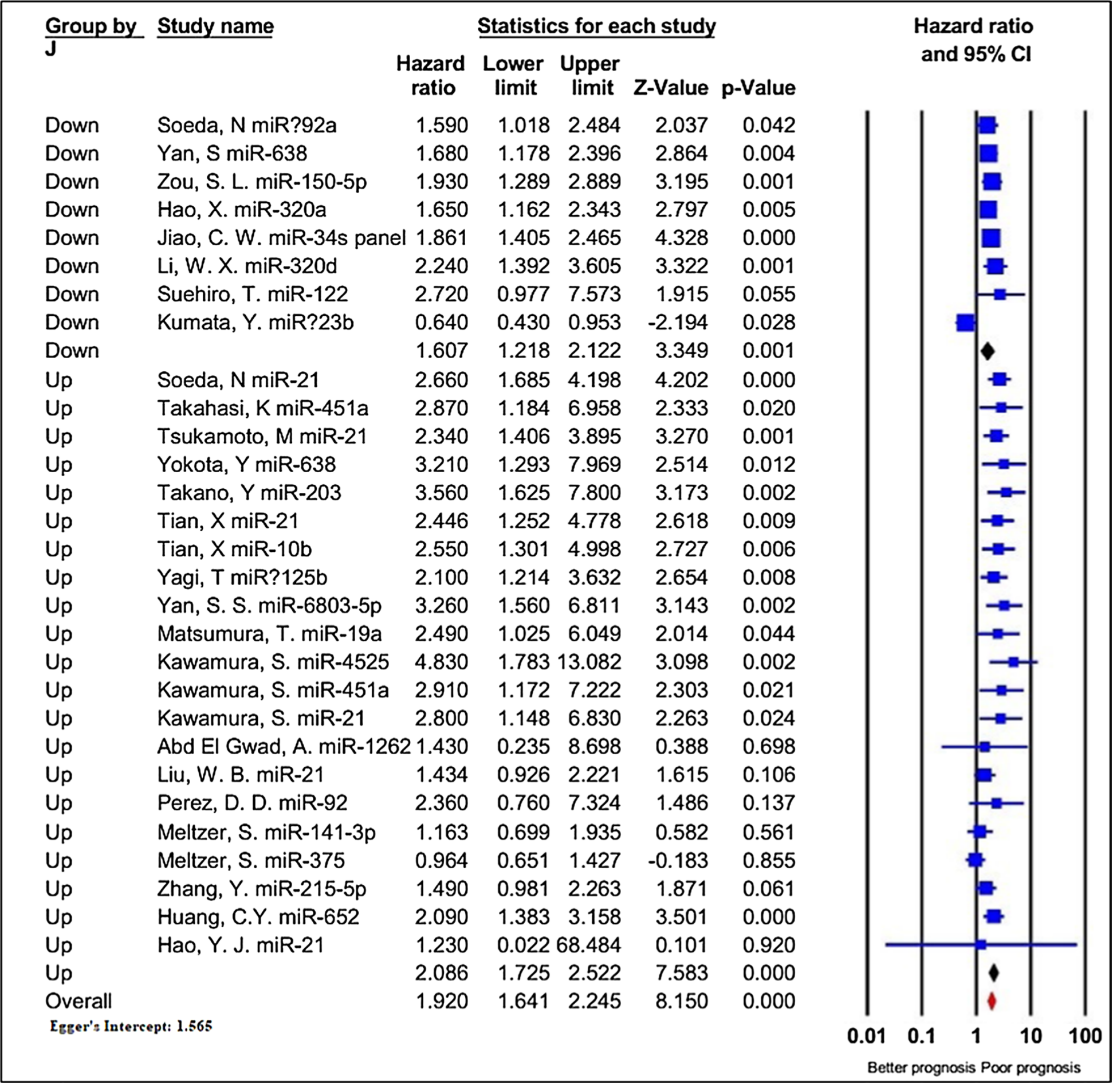
Forest plot of the association between exomiRs deregulation and disease/relapse/progression-free survival (DFS/RFS/PFS) in patients with GI cancers, stratified by the type of exomiRs deregulation (down-regulated exomiRs and up-regulated exomiRs)

As indicated in Table 2, twenty-nine studies containing 2767 patients reported the data regarding DFS/RFS/PFS, and relatively significant heterogeneity was identified among these investigations (I^2^ = 55.871%, P < 0.001). The pooled results through a random-effects model revealed a significant link between deregulated exomiRs and poorer DFS/RFS/PFS in patients (HR = 1.920, 95% CI 1.641–2.245, P < 0.001, Fig. 5).

Following that, subgroup analyses were performed to further investigate the potential predictive significance of the SMYD family members according to the same seven categories utilized for OS (Table 2). The results demonstrated a remarkable connection between deregulated exomiRs and worse DFS/RFS/PFS in all the stratified analyses performed, except patients with GC (HR = 1.408, 95% CI 0.781–2.539, P = 0.256; I^2^ = 86.773%, P < 0.001), LARC (HR = 1.034, 95% CI 0.758–1.410, P = 0.834; I^2^ = 0.000%, P = 0.567), and articles published in Caucasian (HR = 1.109, 95% CI 0.800–1.538, P = 0.535; I^2^ = 10.141%, P = 0.329) (Table 2).

#### Association between deregulated exomiRs and clinicopathological characteristics

##### Gender

The relationship between up-regulated exomiRs and gender was evaluated in 27 studies with 2479 patients, while down-regulated exomiRs were reported in 13 studies with 1736 patients. As shown in Additional file 5: Table S3, pooled results from fixed-effects framework (I^2^ = 19.601%, P = 0.182) indicated that up-regulated exomiRs did not associate with gender (OR = 0.992, 95% CI 0.826–1.190, P = 0.927), as in down-regulated exomiRs (OR = 0.986, 95% CI 0.796–1.222, P = 0.900; I^2^ = 0.000%, P = 0.826).

##### TNM stage

**Fig. 6:**
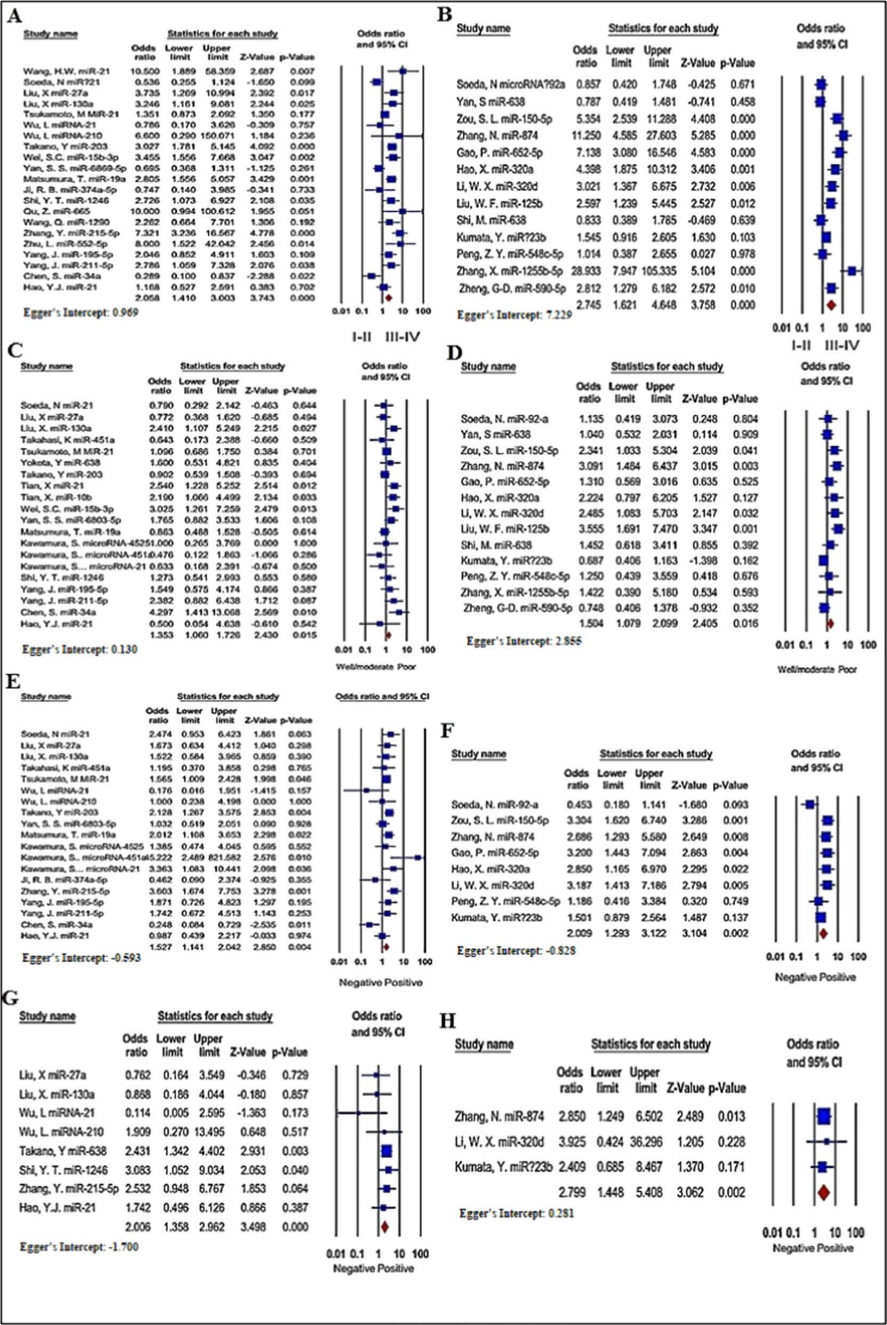
Forest plot of the association between up-regulated **A**, **C**, **E**, and **G** or down-regulated **B**, **D**, **F**, and **H** exomiRs and clinicopathological characteristics in patients with GI cancers. TNM stage **A**, **B**, Differentiation **C**, **D**, Lymph node metastasis **E**, **F**, Distant metastasis **G**, **H**

Data from 21 and 13 investigations comprising of 2288 and 1735 patients were collected and pooled to reveal a connection between up- and down-regulated exomiRs and TNM stage, respectively. Based on the random-effect framework (I^2^ = 71.244%, P < 0.001), it was found that the GI cancer patients with up-regulated exomiRs tended towards the advanced TNM stage (OR = 2.058, 95% CI 1.410–3.003, P < 0.001, Fig. 6A, Additional file 5: Table S3). In addition, the pooled OR indicated that down-regulated exomiRs were directly correlated with a higher TNM stage (HR = 2.745, 95% CI 1.621–4.648, P < 0.001, Fig. 6B, Additional file 5: Table S3).

##### Differentiation

A total of 20 studies 2,425 patients, evaluated a possible connection between up-regulated exomiRs and differentiation (Fig. 6C and Additional file 5: Table S3). The pooled OR through a random effects model (I^2^ = 37.862%, P = 0.045) found statistically significant results (OR = 1.353, 95% CI: 1.060–1.726, P = 0.015). Analysis based on 13 studies through a random-effects model (I^2^ = 55.128%, P = 0.008) showed that the down-regulated exomiRs correspondent with poorly-differentiated cancer cells (OR = 1.504, 95% CI: 1.079–2.099, P = 0.016; Fig. 6D and Additional file 5: Table S3).

##### Lymph node metastasis

A total of 19 studies consisting of 2121 cases focused on the dependability between the up-regulated exomiRs and LNM. The overall pooled HR, under a random-effect model (I^2^ = 47.603%, P = 0.011), indicated that up-regulated exomiRs had a statistically significant relationship with LNM (OR = 1.527, 95% CI 1.141–2.042, P = 0.004; Fig. 6E, Additional file 5: Table S3). Afterward, a clear association was identified between down-regulated exomiRs and LNM (OR = 2.009, 95% CI 1.293–3.122, P = 0.002; Fig. 5F, Additional file 5: Table S3) with significant heterogeneity (I^2^ = 60.459%, P = 0.013).

##### Distant metastasis

The relationship between up-regulated exomiRs and distant metastasis was demonstrated in eight studies with 876 cases, while down-regulated exomiRs were reported in three studies with 467 patients. Statistically, non-significant heterogeneity was observed for high expression exomiRs (I^2^ = 2.450%, P = 0.411) and low expression exomiRs (I^2^ = 0.000%, P = 0.930); consequently, a fixed-effect model was employed to combine the results. The findings showed that cases with down-regulated exomiRs were more likely to develop distant metastasis (OR = 2.799, 95% CI 1.448–5.408, P = 0.002; Fig. 6H, Additional file 5: Table S3), as in up-regulated exomiRs (OR = 2.006, 95% CI 1.358–2.962, P < 0.001; Fig. 6G, Additional file 5: Table S3).

#### Publication Bias

**Fig. 7:**
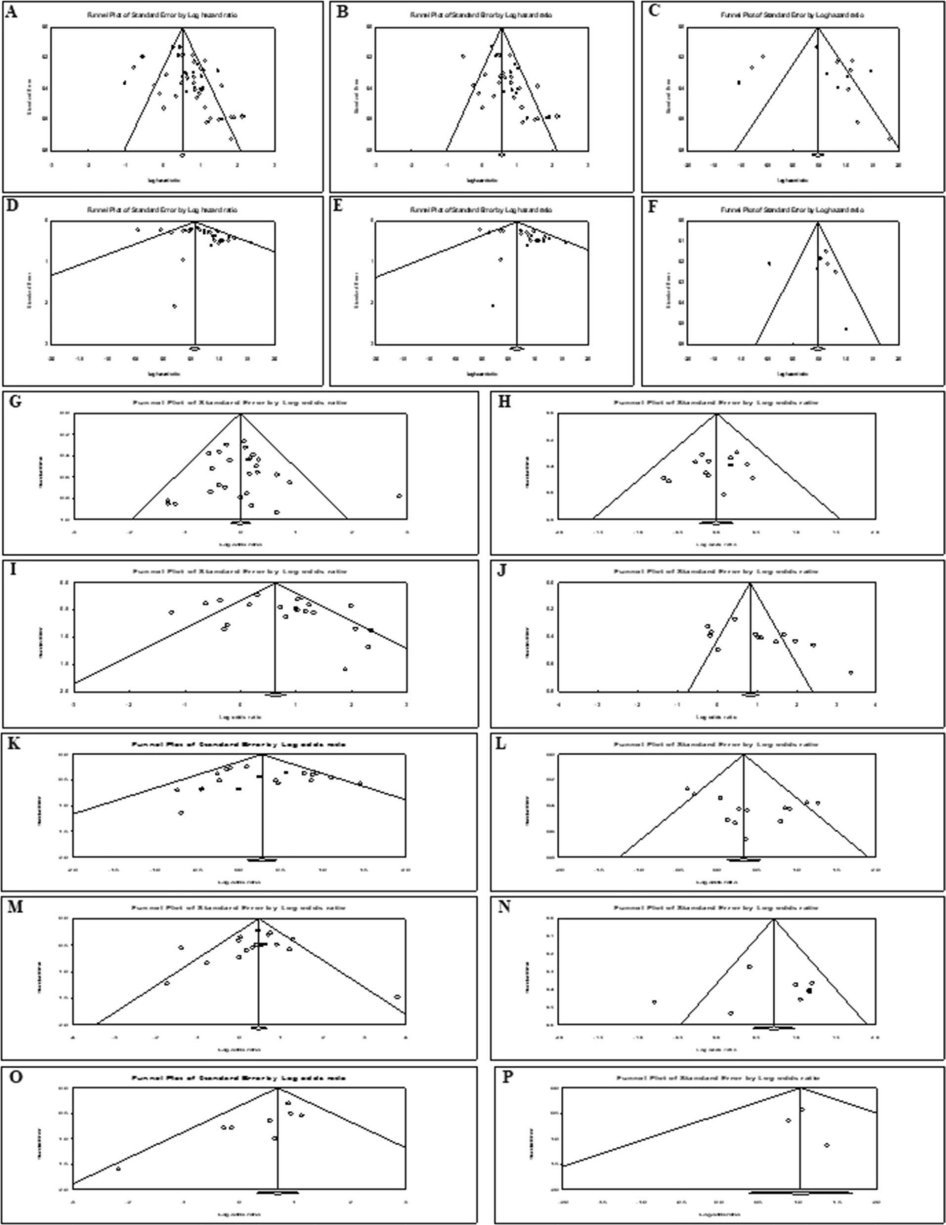
Funnel plot of the publication bias. **A** the association between overall exomiRs deregulation and OS. **B**, **C** the association between up-regulated **B** and down-regulated **C** exomiRs and OS. **D** the association between overall exomiRs deregulation and disease/relapse/progression-free survival (DFS/RFS/PFS). **E**, **F** the association between up-regulated **E** and down-regulated **F** exomiRs and DFS/RFS/PFS. **G**–**P** the association between up-regulated and down-regulated exomiRs and clinicopathological characteristics, including gender **G**, **H**, TNM stage **I**, **J**, Differentiation **K**, **L**, Lymph node metastasis **M**, **N**, Distant metastasis **O**, **P**, respectively

Funnel plot (Fig. 7) and Egger’s test (Additional file 5: Table S3) were also conducted to identify potential publication bias of all analyses. The P-values for Egger’s test, measuring the asymmetry of performed analyses, indicated statistically non-significant publication bias for all analyses except for OS (overall deregulated exomiRs (P = 0.002) and up-regulated exomiRs (P < 0.001), DFS/RFS/PFS (overall deregulated exomiRs (P = 0.021) and up-regulated exomiRs (P = 0.023), TNM stage (down-regulated exomiRs (P = 0.027) and distant metastasis (up-regulated exomiRs (P = 0.026). The funnel plots, displaying potential publication bias, are shown in Fig. 7.

#### Association between circulating exomiR-21 and prognostic/clinicopathological characteristics

To analyze the same types of circulating exomiRs in GI cancers, we considered the exomiR-21 as the most overexpressed miRNA in GI tumors. The results of our analysis indicated a statistically significant association between the exomiR-21 and overall survival (HR = 2.655, 95% CI 1.802–3.912, P < 0.001), disease/relapse/progression-free survival (HR = 2.127, 95% CI 1.674–2.703, P < 0.001), and Lymph node metastasis (HR = 1.568, 95% CI 1.118–2.198, P = 0.009) ( Additional file 2: Figure S2). Furthermore, no association was found between circulating exomiR-21 and other clinicopathological characteristics.

## Discussion

Exploring novel prognostic biomarkers is crucial for improving management, therapy, and prognosis in patients with GI cancers. Compelling evidence has cleared that deregulated exomiRs, as the hallmarks of most human cancers, regulate a plethora of biological pathways, including maintaining proliferative potential, escaping suppressors, repelling cell death, and triggering invasion metastasis, drug resistance, and angiogenesis [80].

Recently, serum exomiRs were applied as feasible and non-invasive serological biomarkers for several cancers, including GI cancers [41, 48, 81–88]. Therefore, in this systematic review and meta-analysis, we aimed to evaluate the prognostic capacity of circulating exomiRs GI cancers. The four principal databases (Web of Science, PubMed/MEDLINE, Scopus, and Embase) were searched to ensure the identification of all relevant studies. To the best of our knowledge, this is the first comprehensive meta-analysis comprising a wide range of circulating exomiRs with 4881 participants to provide strong evidence regarding the potential clinical applicability of circulating exomiRs in GI cancers. Notably, we attempted to clarify study heterogeneity and publication bias.

In general, we combined the data regarding the deregulation of all exomiRs in our meta-analysis to perceive their potential roles to predict the prognosis. The results emerging from this meta-analysis advocated that deregulated exomiRs were significantly associated with reduced OS (HR = 1.998) and dismal DFS/RFS/PFS (HR = 1.920), supporting the statement that the aberrant expression of exomiRs as minimally invasive biomarkers can predict prognostic outcomes in GI cancers patients. The majority of subcategories, including the type of exomiRs deregulation (up or down), the majority of cancer types (colorectal, hepatocellular, and pancreatic cancers), sample size (≥ 100 or < 100), various data extraction methods (direct or indirect), sample type (plasma or serum), and NOS score (high or low-quality publications), were also strongly associated with poor survival outcomes (OS or DFS/RFS/PFS, strengthening the prognosis. Only some types of cancers (gastric cancer and locally advanced rectal cancer) and Caucasian ethnicity did not show a link with poor survival outcomes. Based on obtained findings from OS, CRC (HR = 2.928), HCC (HR = 1.582), and PDAC (HR = 2.514) are likely to have a significant association with poor prognosis while other cancers of the GI tract, including GC (HR = 1.353, P = 0.229) and LARC (HR = 0.958, P = 0.857) did not show such association. Even the association between DFS/RFS/PFS and the same types of cancers including CRC (HR = 2.105), HCC (HR = 1.923), and PDAC (HR = 3.195) remained significant. This finding highlights the similarity of results related to different types of prognostic reports (OS vs. DFS/RFS/PFS) and GI cancers. Moreover, we did not find any significant association between survival findings and LARC or GC. It is assumed that the assorted outcomes and the insufficient number of included studies might have affected these findings [89, 90]. It was reported that variability in outcomes prediction and categorization of LARC is due to the diversity of tumors and the complexity of diagnostic and prognostic tools in clinical practice [91–93].

We observed partially high heterogeneity in most of our prognostic findings, which is mainly due to the considerable variability within circulating exomiRs, type of cancer, and follow-up duration of the included studies. Moreover, publication bias was only detected between OS and overall deregulated exomiRs (P = 0.002) or up-regulated exomiRs (P < 0.001), as well as DFS and overall deregulated exomiRs (P = 0.021) or up-regulated exomiRs (P = 0.023).

The clinical significance of circulating exomiRs with abnormally elevated or reduced expression in various malignancies has been highlighted by an increasing body of research [79, 98, 99]. We examined 47 studies on 62 distinct exomiRs in GI malignancies in the current research, including 50 exomiRs with high expression, 16 with low expression, and 4 with both low and high expression (miR-122, 638, 125b, 92a). All circulating exomiRs were categorized into two main subgroups as either up- or down-regulated exomiRs. According to our findings, aberrant expression of exomiRs, in terms of both up and down-regulation, are strongly associated with inferior prognostic outcomes, including OS and DFS/RFS/PFS, as well as clinicopathological characteristics, including differentiation, TNM stage, lymph node metastasis, and distant metastasis. However, we did not identify any association between up or down-regulated circulating exomiRs and gender. Similar to our findings, a prior meta-analysis has demonstrated that aberrant exomiRs expression, in terms of both up and down-regulation, is correlated with a worse prognosis in colorectal cancer [23]. The above findings propose that developing the panel of exomiRs could be utilized as valuable biomarkers in GI cancer prognosis.

Among the various up-regulated miRs, miR-21 has been identified to be the most overexpressed miRNA in tumors, which may affect the development of cancer via different signaling cascades [94]. Overexpression of miR-21 was recognized as a prognostic and diagnostic biomarker in various cancers [95–99]. Furthermore, it was studied that high expression of miR-21 was correlated with low OS in glioma patients [100]. Our findings, which indicated a statistically significant correlation between the exomiR-21 and OS (HR = 2.655, P < 0.001), DFS/RFS/PFS (HR = 2.127, P < 0.001), and Lymph node metastasis (HR = 1.568, P = 0.009), are consistent with these findings. Similarly, overexpression of miR-222 can enhance tumorigenesis, migration, and invasion properties in breast cancer (BC) and thyroid cancer [101, 102], along with its association with worse OS and DFS/RFS/PFS in glioma and NSCLC [103, 104]. Besides, high expression of the miR-200 family plays a significant role in tumorigenesis and metastasis in ovarian cancer and endometrial adenocarcinoma [105, 106] and is associated with shorter OS in breast cancer [107]. Regarding the results of our meta-analysis and other investigations, overexpression of some specific exomiRs has the chance to become predictors of long-term survival and metastasis in the patients with GI cancers.

Regarding down-regulated miRs, a low level of miR-320 is correlated with advanced stage, LNM, and poor OS in BC patients [108]. It was reported that miR-34 is expressed at low levels in BC, NSCLC, and bladder cancer and its down-regulation is correlated with recurrence, metastasis, and poor survival outcomes [109–111]. Furthermore, the down-regulation of miR-30c was correlated with poor prognostic outcomes in patients with BC [112, 113]. Thus, our findings revealed that up- or down-regulated exomiRs can be considered new biomarkers in predicting clinical outcomes in GI cancers.

Moreover, the expression of some circulating exomiRs could be either increased or decreased based on the cancer type. Among them, miR-122, a tumor suppressor miR, could regulate metastasis of HCC [114], and circulating miR-122 was up-regulated in BC, NSCLC [115, 116], which is associated with distant metastasis and lowered OS and PFS, considered as a prognostic factor [115]. Other groups of researchers, however, have shown that miR-122 expression is down-regulated in various cancer cells, including bladder and colon tumors [105]. These investigations support our findings, which show that several circulating exomiRs (miR-122, 638, 125b, and 92a) are members of both expression-high and expression-low subgroups in GI cancers. Although our large meta-analysis sheds light on the pathological characteristics and prognostic potentials of GI circulating exomiRs in clinical practice, several limitations should be considered when interpreting the results of the current study. Firstly, while all relevant studies were included in this study, relatively high heterogeneity was observed when analyzing the overall findings for OS or DFS/RFS/PFS. Therefore, subgroup analysis was performed by considering some subcategories to find the factors that caused the heterogeneity. Regarding this consideration, we did not underestimate the variability in patients’ clinicopathological characteristics. Therefore, variations in population and research methodology might affect the heterogeneity. Secondly, the publication bias was identified for the relationship between OS or DFS/RFS/PFS and circulating exomiRs with high expression, affecting the validity of prognostic findings. Third, additional bias may possibly have arisen from (i) the number of investigations for some GI cancers was insufficient; (ii) all articles published in English; and (iii) most of the included publications were performed in Asian ethnicity, possibly leading to selection bias. Finally, some statistical errors may affect the credibility of findings in our meta-analysis, including (i) the indirect estimation of HR and 95% CI via the Kaplan–Meier curves in some studies; and (ii) using univariate analysis information instead of multivariate analysis for some studies without providing the statistical methodology. Despite these limitations, this meta-analysis suggests that circulating exomiRs would be useful as new prognostic biomarkers in GI cancers. However, multi-parameter and large-scale clinical studies with a strong methodology are required to implement exomiRs as biomarkers in the prognosis of GI cancers robustly.

## Conclusions

In conclusion, our study demonstrated the widest meta-analysis conducted on the prognostic importance of circulating exomiRs in GI cancers. Our results indicated that up- and down-regulated circulating exomiRs might serve as effective indicators of inferior survival outcomes in patients with gastrointestinal malignancies. In addition, exomiR dysregulation is related to advanced clinical stage, poor differentiation, and tumor spread in GI carcinomas. The current review advocates using a combined panel of circulating exomiRs for better risk stratification and clinical outcomes prediction in GI cancer patients, which could compensate for the unreliability of individual exomiRs in estimating prognosis. We envision our results would bring the attention of researchers and clinicians to the significance of deregulated circulating exomiRs as prognostic biomarkers that may aid better prediction.

## Supplementary Information

**Additional file 1: Figure S1.** Forest plot of the association between NOS and overall survival (A), disease/relapse/progression-free survival (B).

**Additional file 2: Figure S2.** Forest plot of the association between exomiR-21 and overall survival (A), disease/relapse/progression-free survival (B), and clinicopathological characteristics in patients with GI cancers. Lymph node metastasis (C), Differentiation (D), Distant metastasis (E), TNM stage (F), Gender (G).

**Additional file 3: Table S1.** Search strategy of electronical databases.

**Additional file 4: Table S2.** Significantly dysregulated circulating exomiR(s) in GI cancer patients.

**Additional file 5: Table S3.** Summary of meta-analyses for the association between exomiRs deregulation and clinicopathologic features in patients with GI cancers.

## Data Availability

All recorded data from the data extraction process were available on request to the extent that they were not included in the systematic review article.
