# Supplementary material for: Circulating exosomal microRNAs as potential prognostic biomarkers in gastrointestinal cancers: a systematic review and meta-analysis
Source: Cancer Cell Int. 2023 Jan 20;23:10. doi: 10.1186/s12935-023-02851-8 (PMC9862982; doi:10.1186/s12935-023-02851-8)
Supplement: Supplementary file 1 — Additional file 1: Figure S1. Forest plot of the association between NOS and overall survival (A), disease/relapse/progression-free survival (B). [file 12935_2023_2851_MOESM1_ESM.docx]

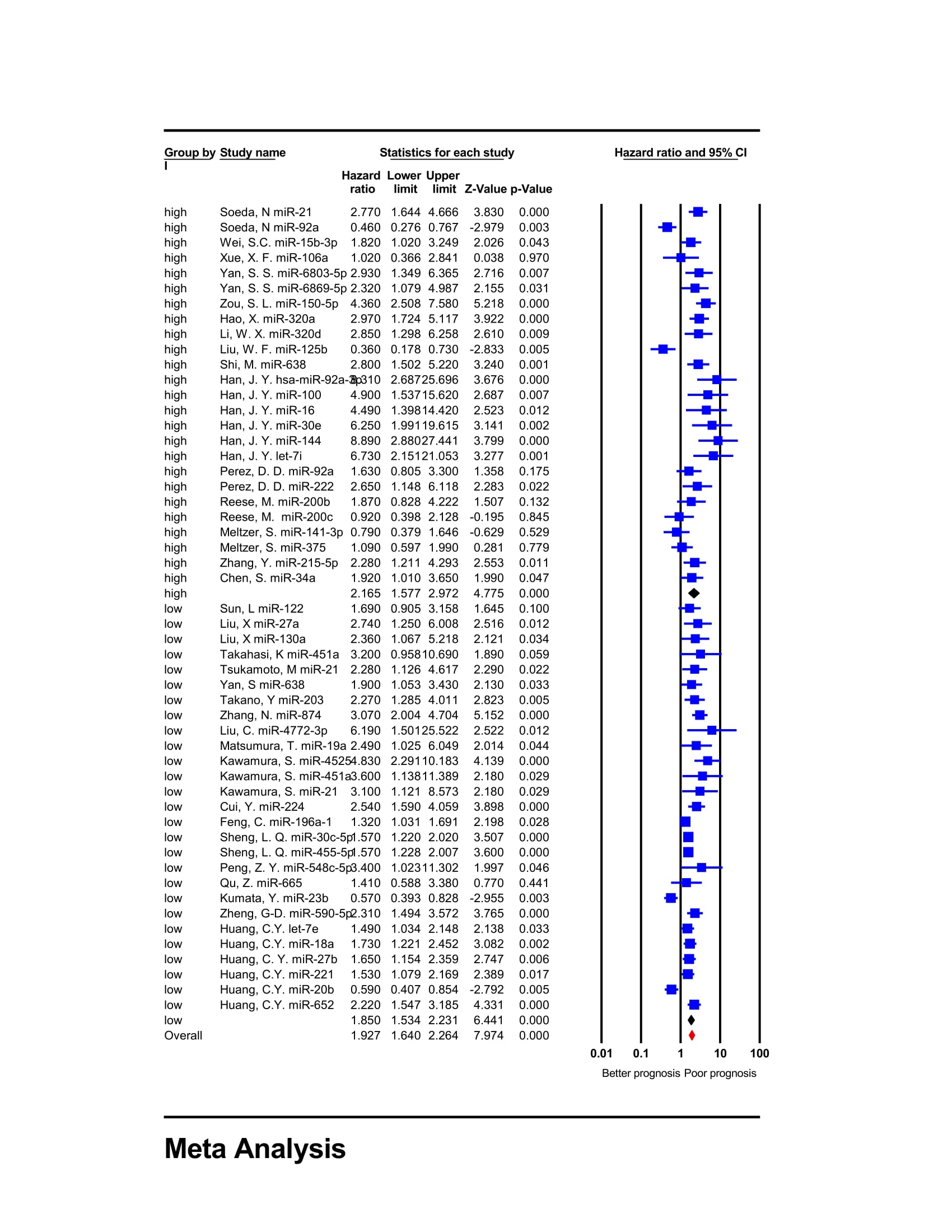


**A**


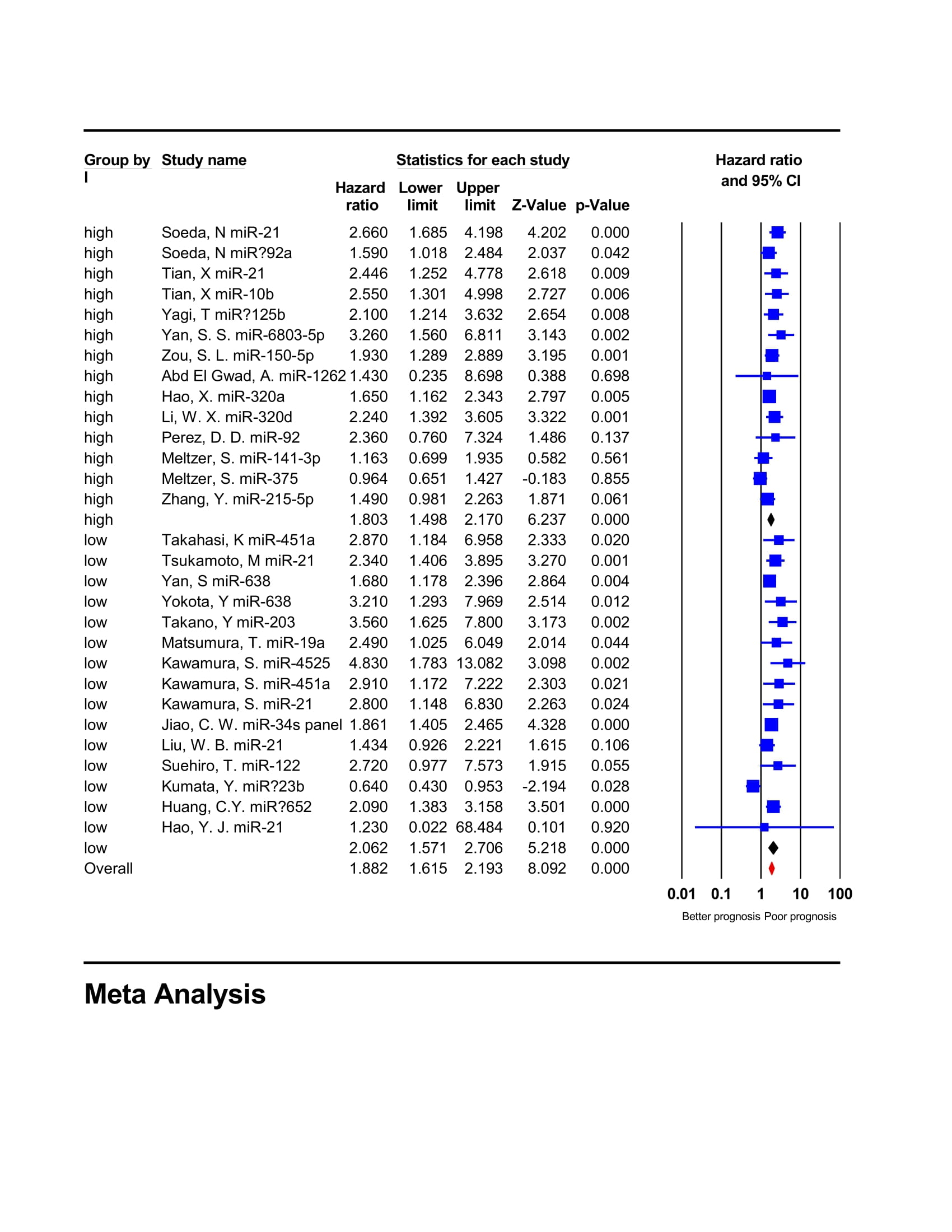


**B**

**Figure S1.** Forest plot of the association between NOS and overall survival (A), disease/relapse/progression-free survival (B).
