# Supplementary material for: Circulating exosomal microRNAs as potential prognostic biomarkers in gastrointestinal cancers: a systematic review and meta-analysis
Source: Cancer Cell Int. 2023 Jan 20;23:10. doi: 10.1186/s12935-023-02851-8 (PMC9862982; doi:10.1186/s12935-023-02851-8)
Supplement: Supplementary file 2 — Additional file 2: Figure S2. Forest plot of the association between exomiR-21 and overall survival (A), disease/relapse/progression-free survival (B), and clinicopathological characteristics in patients with GI cancers. Lymph node metastasis (C), Differentiation (D), Distant metastasis (E), TNM stage (F), Gender (G). [file 12935_2023_2851_MOESM2_ESM.docx]

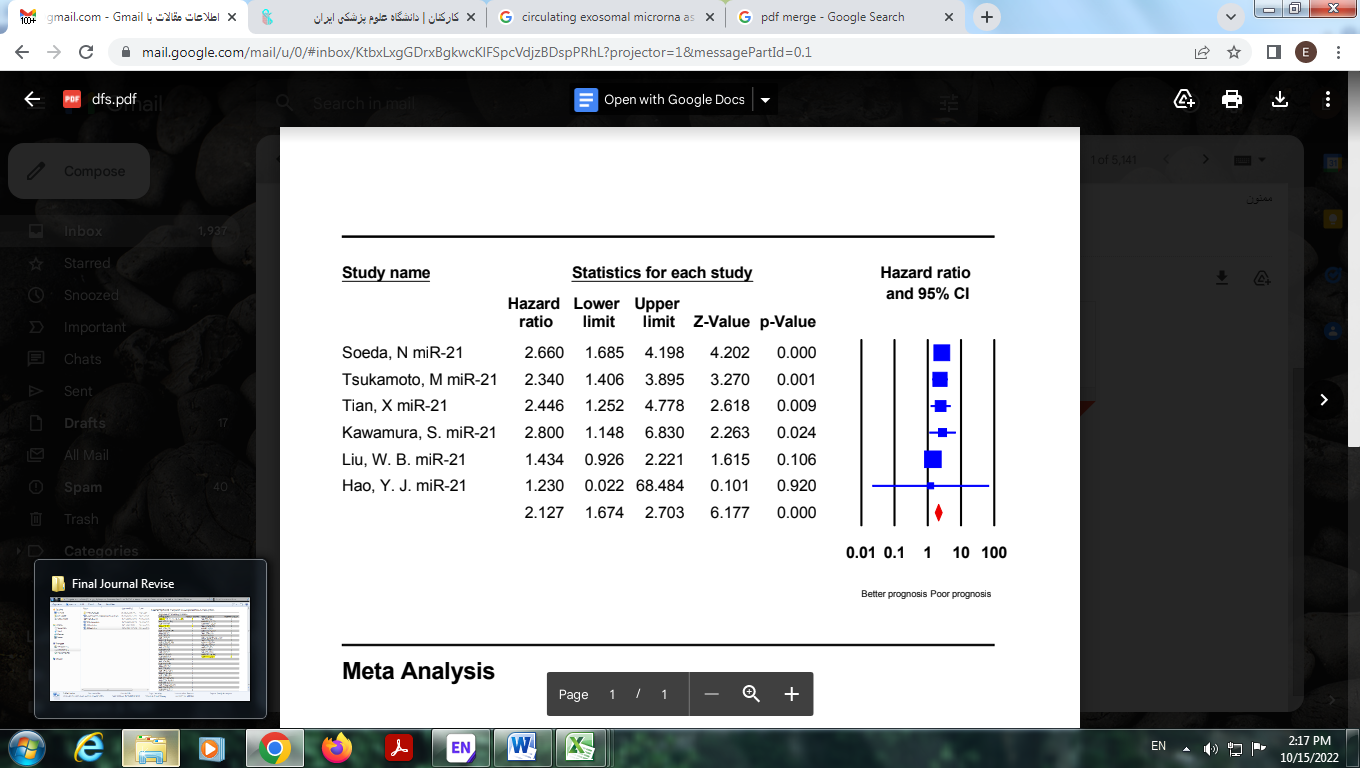


**A**


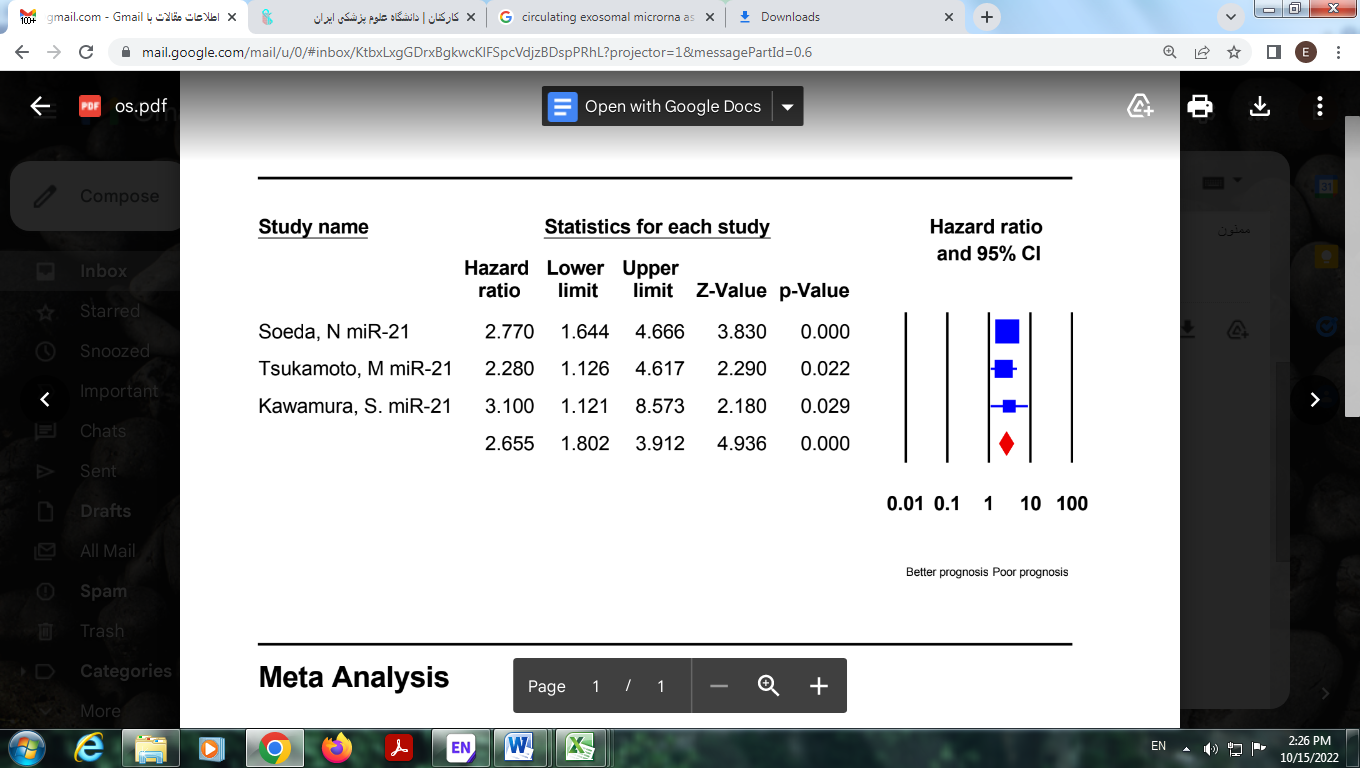

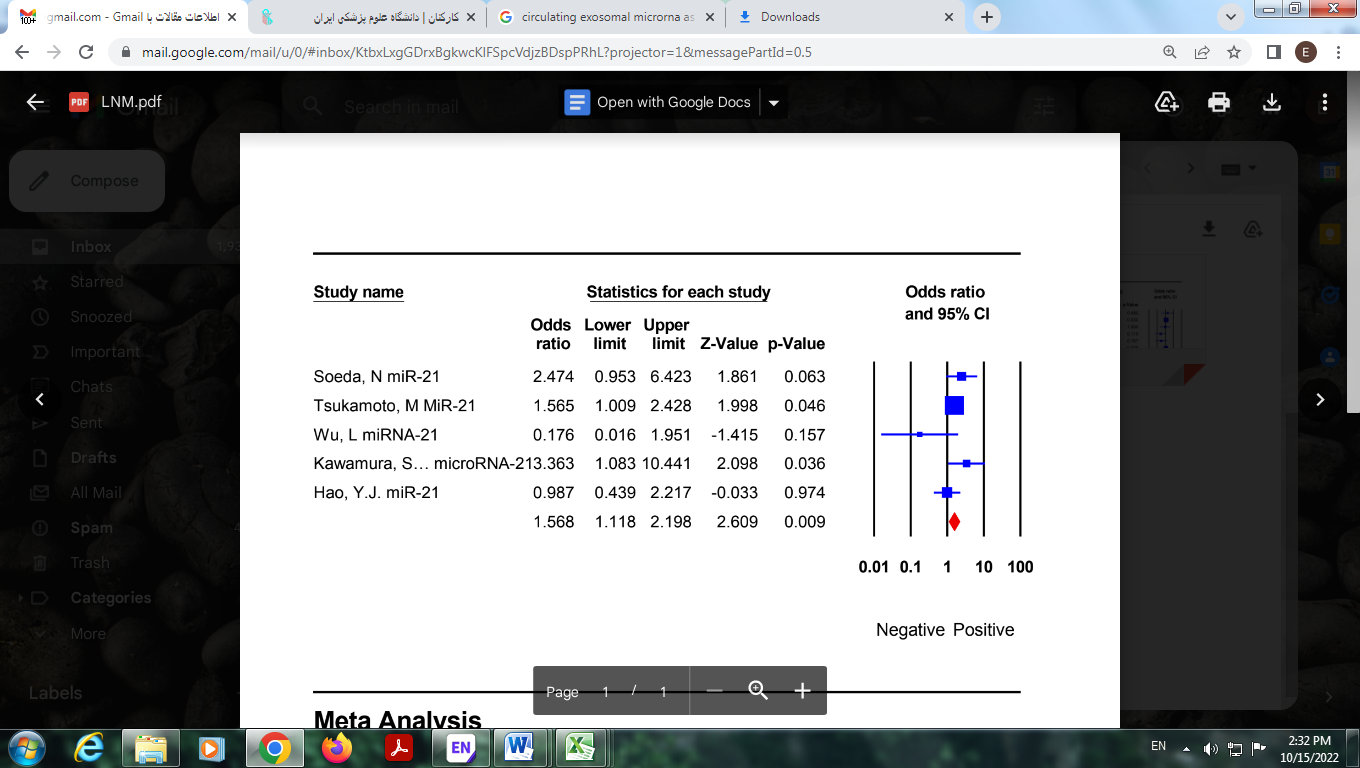

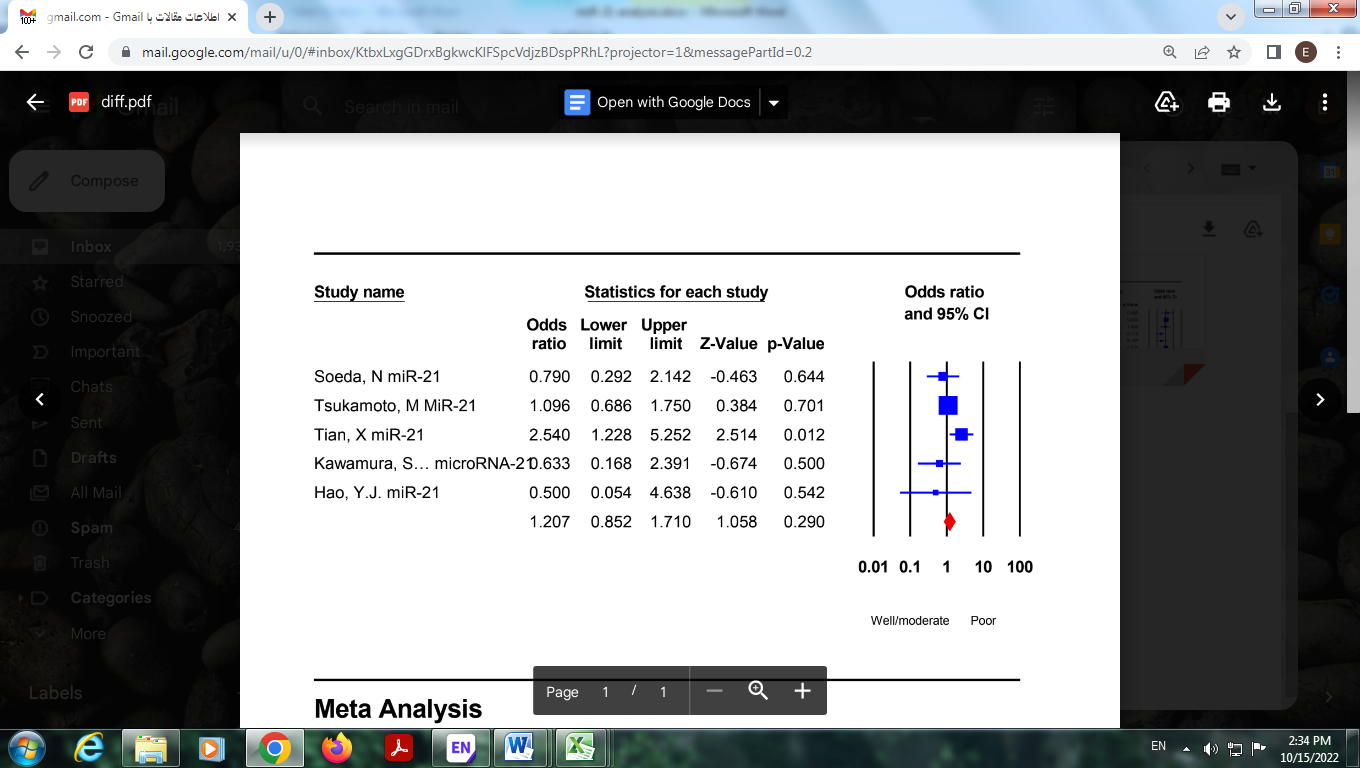

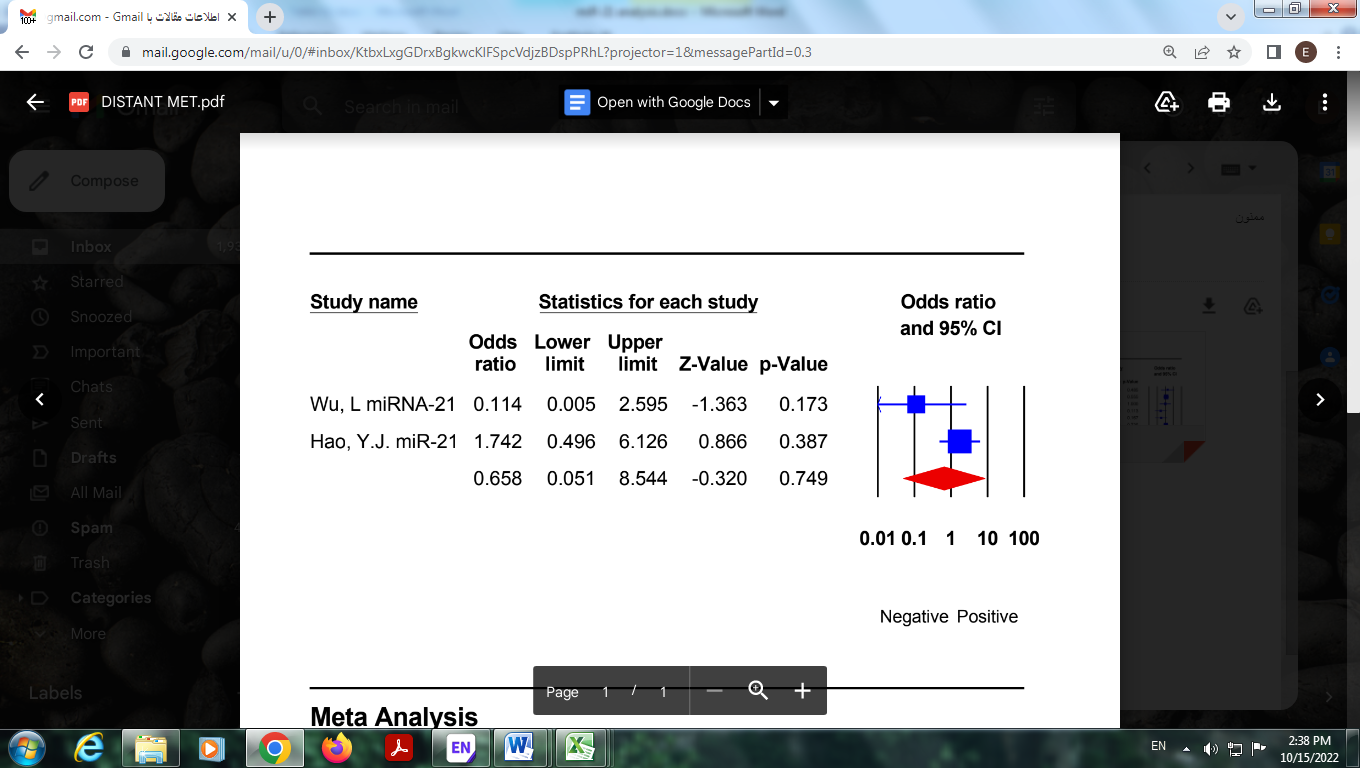

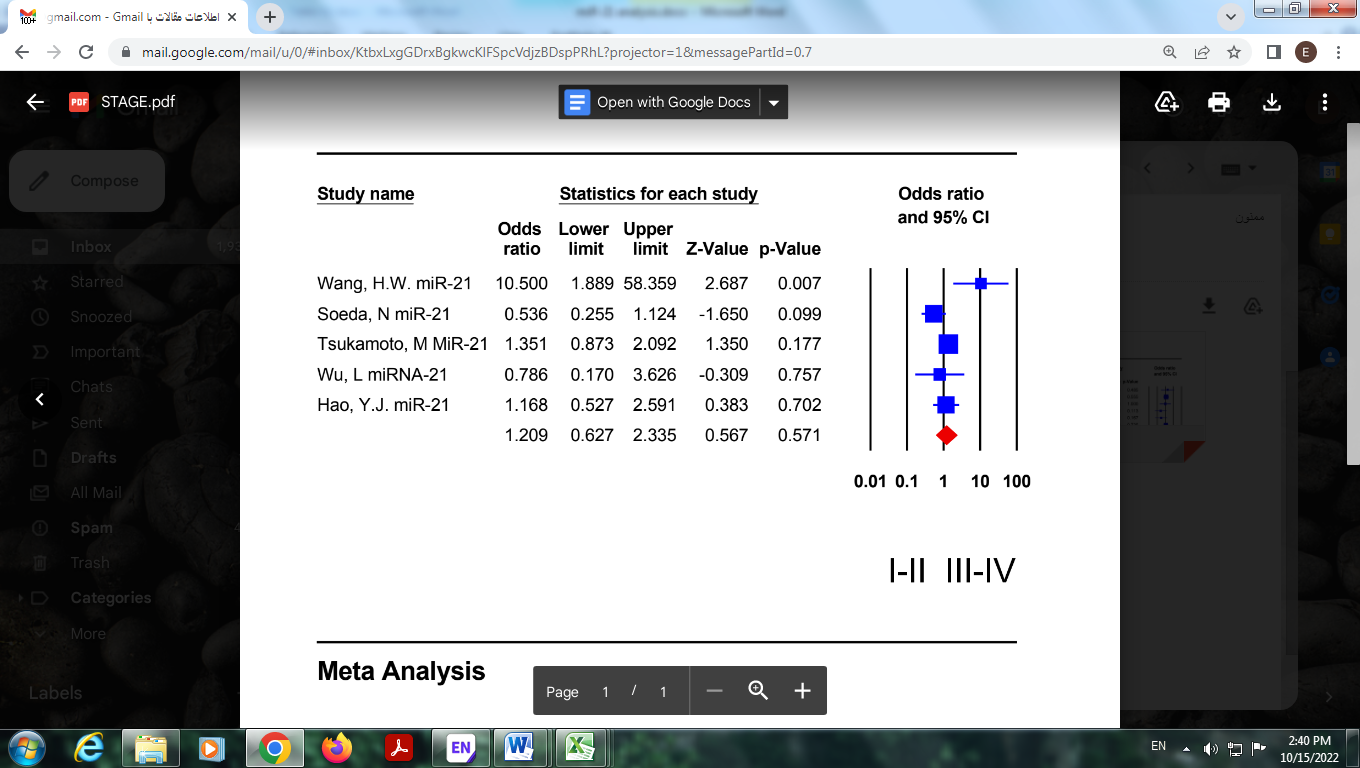

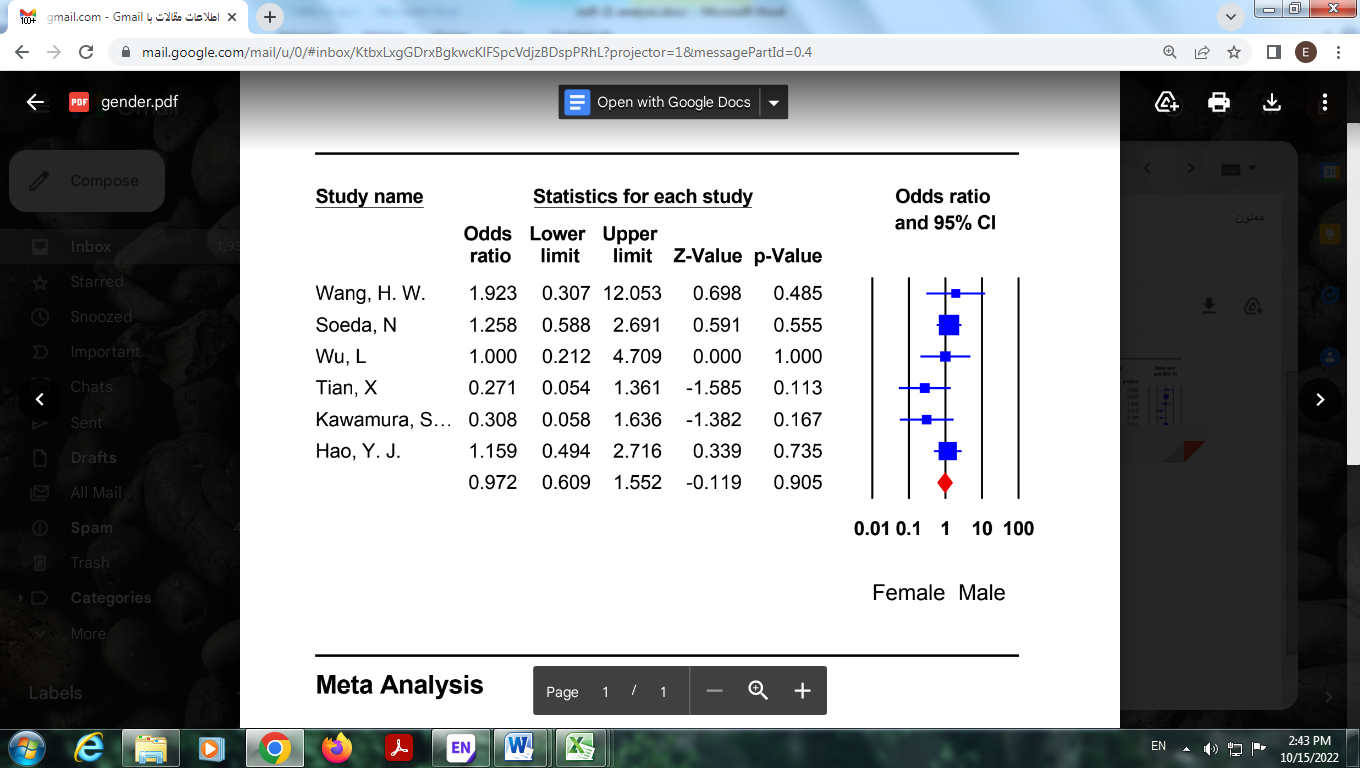


**B**

**C**

**D**

**E**

**F**

**G**

**Figure S2.** Forest plot of the association between exomiR-21 and overall survival (A), disease/relapse/progression-free survival (B), and clinicopathological characteristics in patients with GI cancers. Lymph node metastasis (C), Differentiation (D), Distant metastasis (E), TNM stage (F), Gender (G).
