# Supplementary material for: Circulating exosomal microRNAs as potential prognostic biomarkers in gastrointestinal cancers: a systematic review and meta-analysis
Source: Cancer Cell Int. 2023 Jan 20;23:10. doi: 10.1186/s12935-023-02851-8 (PMC9862982; doi:10.1186/s12935-023-02851-8)
Supplement: Supplementary file 3 — Additional file 3: Table S1. Search strategy of electronical databases. [file 12935_2023_2851_MOESM3_ESM.docx]

**Table S1.** Search strategy of electronical databases.

| **PubMed** | **Total Number of Papers** |
| --- | --- |
| mesentery[Mesh] OR stomach[Mesh] OR Pancreas[Mesh] OR rectum[Mesh] OR duodenum[Mesh] OR jejunum[Mesh] OR ileum[Mesh] OR cecum[Mesh] OR colon[Mesh] OR mouth[Mesh] OR esophagus[Mesh] OR Gastrointestinal Tracts[Mesh] OR "Gastrointestinal Tracts"[tiab] OR "GI Tract"[tiab] OR "GI Tracts"[tiab] OR "gastrointestinal tract"[tiab] OR "Digestive Tract"[tiab] OR "Digestive Tracts"[tiab] OR "Lower GI Tract"[tiab] OR GI[tiab] OR oral[tiab] OR ‎mouth[tiab] OR esophagus[tiab] OR gullet[tiab] OR gastric[tiab] OR duodenum[tiab] OR jejunum[tiab] OR ileum[tiab] OR cecum[tiab] OR colon[tiab] OR colorectal[tiab] OR sigmoid[tiab] OR ‎rectum[tiab] OR anus[tiab] OR mesentery[tiab]‎ OR hepatic[tiab] OR liver[tiab] OR hepatocellular[tiab] OR stomach[tiab] OR Pancrea*[tiab] |  |
| Neoplasms[Mesh] OR Cancer*[tiab] OR Neoplasm*[tiab] OR ‎Carcinoma[tiab] OR Tumo*[tiab] |  |
| extracellular vesicles[Mesh] OR exosomes[Mesh] OR exosome*[tiab] OR "extracellular ‎vesicle"[tiab] OR "extracellular vesicles"[tiab] OR microvesicle[tiab] OR "Shedding Microvesicles"[tiab]‎ |  |
| ‎ #1 AND #2 AND #3 | 3,120 |
| **Web of Sciences** | |
| TOPIC: (mesentery OR stomach OR rectum OR duodenum OR jejunum OR ileum OR cecum OR colon OR mouth OR esophagus OR "Gastrointestinal Tracts" OR "GI Tract" OR "GI Tracts" OR "Digestive Tract" OR "Digestive Tracts" OR "Lower GI Tract" OR GI OR oral OR gullet OR gastric OR colorectal OR sigmoid OR anus‎ OR hepatic OR liver OR hepatocellular OR Pancrea*) |  |
| TOPIC: (Cancer* OR Neoplasm* OR ‎Carcinoma OR Tumo*) |  |
| TOPIC: (exosome* OR "extracellular ‎vesicle" OR "extracellular vesicles" OR microvesicle OR "Shedding Microvesicles"‎) |  |
| ‎ #1 AND #2 AND #3 | 4,438 |
| **SCOPUS** | |
| ‎ TITLE-ABS-KEY (mesentery OR stomach OR rectum OR duodenum OR jejunum OR ileum OR cecum OR colon OR mouth OR esophagus OR "Gastrointestinal Tracts" OR "GI Tract" OR "GI Tracts" OR "Digestive Tract" OR "Digestive Tracts" OR "Lower GI Tract" OR GI OR oral OR gullet OR gastric OR colorectal OR sigmoid OR anus‎ OR hepatic OR liver OR hepatocellular OR Pancrea*) |  |
| ‎‎ TITLE-ABS-KEY (Cancer* OR Neoplasm* OR ‎Carcinoma OR Tumo*) |  |
| ‎‎TITLE-ABS-KEY (exosome* OR "extracellular ‎vesicle" OR "extracellular vesicles" OR microvesicle OR "Shedding Microvesicles"‎) |  |
| #1 AND #2 AND #3 | 5,186 |
| **Embase** | |
| mesentery:ab,ti OR stomach:ab,ti OR rectum:ab,ti OR duodenum:ab,ti OR jejunum:ab,ti OR ileum:ab,ti OR cecum:ab,ti OR colon:ab,ti OR mouth:ab,ti OR esophagus:ab,ti OR "Gastrointestinal Tracts":ab,ti OR "GI Tract":ab,ti OR "GI Tracts":ab,ti OR "Digestive Tract":ab,ti OR "Digestive Tracts":ab,ti OR "Lower GI Tract":ab,ti OR GI:ab,ti OR oral:ab,ti OR gullet:ab,ti OR gastric:ab,ti OR colorectal:ab,ti OR sigmoid:ab,ti OR anus:ab,ti ‎ OR hepatic:ab,ti OR liver:ab,ti OR hepatocellular:ab,ti OR Pancrea*:ab,ti |  |
| Cancer*:ab,ti OR Neoplasm*:ab,ti OR ‎Carcinoma:ab,ti OR Tumo*:ab,ti |  |
| exosome*:ab,ti OR "extracellular ‎vesicle":ab,ti OR "extracellular vesicles":ab,ti OR microvesicle:ab,ti OR "Shedding Microvesicles":ab,ti ‎ |  |
| ‎#1 AND #2 AND #3 | 3,988 |
