# Supplementary material for: Circulating exosomal microRNAs as potential prognostic biomarkers in gastrointestinal cancers: a systematic review and meta-analysis
Source: Cancer Cell Int. 2023 Jan 20;23:10. doi: 10.1186/s12935-023-02851-8 (PMC9862982; doi:10.1186/s12935-023-02851-8)
Supplement: Supplementary file 4 — Additional file 4: Table S2. Significantly dysregulated circulating exomiR(s) in GI cancer patients. [file 12935_2023_2851_MOESM4_ESM.docx]

**Table S2.** Significantly dysregulated circulating exomiR(s) in GI cancer patients.

| Expression of circulating exomiR(s) | | | |
| --- | --- | --- | --- |
| Upregulated | **Number of study** | **Downregulated** | **Number of study** |
| miR-21 (37, 40, 42, 45, 51, 55, 68) | 6 | miR-92a (37) | 1 |
| miR-122 (38, 47) | 2 | miR-638 (69, 79) | 2 |
| miR-27a (39) | 1 | miR-6869-5p (70) | 1 |
| miR-27b (67) | 1 | miR-150-5p (71) | 1 |
| miR-130a (39) | 1 | miR-874 (72) | 1 |
| miR-451a (41, 51) | 2 | miR-4772-3p (73) | 1 |
| miR-638 (43) | 1 | miR-320a (74) | 1 |
| miR-203 (44) | 1 | miR-320d (76) | 1 |
| miR-10b (45) | 1 | miR-34s panel (a,b,c) (75) | 1 |
| miR-15b-3p (46) | 1 | miR-125b (77) | 1 |
| miR-125b (47, 48) | 2 | miR-455-5p (78) | 1 |
| miR-145 (47) | 1 | miR-30c-5p (78) | 1 |
| miR-192 (47) | 1 | miR-122 (80) | 1 |
| miR-194 (47) | 1 | miR-23b (122) | 1 |
| miR-29a (47) | 1 | miR-548c-5p (82) | 1 |
| miR-17-5p (47) | 1 | miR-590-5p (83) | 1 |
| miR-106a (47) | 1 |  |  |
| miR-6803-5p (49) | 1 |  |  |
| miR-19a (50) | 1 |  |  |
| miR-4525 (51) | 1 |  |  |
| miR-1262 (52) | 1 |  |  |
| miR-224 (53) | 1 |  |  |
| miR-196a-1 (54) | 1 |  |  |
| miR-25-3p (56) | 1 |  |  |
| miR-1269a (56) | 1 |  |  |
| miR-4661-5p (56) | 1 |  |  |
| miR-4746-5p (56) | 1 |  |  |
| miR-100-5p (57) | 1 |  |  |
| miR-92a-3p (57, 58) | 2 |  |  |
| miR-30e-5p (57) | 1 |  |  |
| miR-144-5p (57) | 1 |  |  |
| Let-7i-5p (57) | 1 |  |  |
| Let-7e (67) | 1 |  |  |
| miR-16 (57) | 1 |  |  |
| miR-222 (58) | 1 |  |  |
| miR-665 (59) | 1 |  |  |
| miR-200b (60) | 1 |  |  |
| miR-200c (60) | 1 |  |  |
| miR-141-3p (61) | 1 |  |  |
| miR-375 (61) | 1 |  |  |
| miR-1290 (62) | 1 |  |  |
| miR-215-5p (63) | 1 |  |  |
| miR-552-5p (64) | 1 |  |  |
| miR-195-5p (65) | 1 |  |  |
| miR-211-5p (65) | 1 |  |  |
| miR-34a (66) | 1 |  |  |
| miR-18a (67) | 1 |  |  |
| miR-221 (67) | 1 |  |  |
| miR-20b (67) | 1 |  |  |
| miR-652 (67) | 1 |  |  |
