# Supplementary material for: Circulating exosomal microRNAs as potential prognostic biomarkers in gastrointestinal cancers: a systematic review and meta-analysis
Source: Cancer Cell Int. 2023 Jan 20;23:10. doi: 10.1186/s12935-023-02851-8 (PMC9862982; doi:10.1186/s12935-023-02851-8)
Supplement: Supplementary file 5 — Additional file 5: Table S3. Summary of meta-analyses for the association between exomiRs deregulation and clinicopathologic features in patients with GI cancers. [file 12935_2023_2851_MOESM5_ESM.docx]

**Table S3.** Summary of meta-analyses for the association between exomiRs deregulation and clinicopathologic features in patients with GI cancers.

|  | **Study number** | **Sample size** | **Association** | | **Heterogeneity** | | **Publication bias** | |
| --- | --- | --- | --- | --- | --- | --- | --- | --- |
|  |  |  | **OR/HR (95% CI)** | **P value** | **I^2^ (%)** | **P_h_** | **P value** | **t value** |
| **Upregulated exomiRs** | | | | | | | | |
| Gender (male vs. female) | 27 | 2479 | 0.992 (0.826-1.190) | 0.927 | 19.601 | 0.182 | 0.740 | 0.335 |
| TNM stage (III+IV vs. I+II) | 21 | 2288 | 2.058 (1.410-3.003) | < 0.001 | 71.244 | < 0.001 | 0.342 | 0.973 |
| Differentiation (poor vs. well) | 20 | 2425 | 1.353 (1.060-1.726) | 0.015 | 37.862 | 0.045 | 0.880 | 0.152 |
| LNM (positive vs. negative) | 19 | 2121 | 1.527 (1.141-2.042) | 0.004 | 47.603 | 0.011 | 0.474 | 0.731 |
| DM (positive vs. negative) | 8 | 876 | 2.006 (1.358-2.962) | < 0.001 | 2.450 | 0.411 | 0.026 | 2.930 |
| **Downregulated exomiRs** | | | | | | | | |
| Gender (male vs. female) | 13 | 1736 | 0.986 (0.796-1.222) | 0.900 | 0.000 | 0.826 | 0.349 | 0.976 |
| TNM stage (III+IV vs. I+II) | 13 | 1735 | 2.745 (1.621-4.648) | < 0.001 | 83.318 | < 0.001 | 0.027 | 2.544 |
| Differentiation (poor vs. well) | 13 | 1729 | 1.504 (1.079-2.099) | 0.016 | 55.128 | 0.008 | 0.118 | 1.693 |
| LNM (positive vs. negative) | 8 | 1051 | 2.009 (1.293-3.122) | 0.002 | 60.459 | 0.013 | 0.794 | 0.272 |
| DM (positive vs. negative) | 3 | 467 | 2.799 (1.448-5.408) | 0.002 | 0.000 | 0.930 | 0.699 | 0.510 |

LNM: Lymph node metastasis, DM: Distant metastasis
